# Umbilical Cord‐Mesenchymal Stromal Cell‐Derived Extracellular Vesicles Target the Liver to Improve Neurovascular Health in Type 2 Diabetes With Non‐Alcoholic Fatty Liver Disease

**DOI:** 10.1002/jev2.70125

**Published:** 2025-07-07

**Authors:** Minghao Du, Hao Yang, Jinyun Niu, Jing Huang, Lihong Wang, Junxiu Xi, Panpan Meng, Zhiyong Liu, Guaiguai Ma, Jiani Li, Xiaoyan Liu, Liang Guo, Mingjun Hu, Zhufang Tian, Bin Liu, Weiping Liu, Ashok K. Shetty, Shengxi Wu, Andrius Baskys, Qianfa Long

**Affiliations:** ^1^ Department of Neurosurgery, Xi'an Central Hospital Xi'an Jiaotong University Xi'an China; ^2^ Key Laboratory of Central Nervous System Diseases Xi'an Jiaotong University Xi'an China; ^3^ Key Laboratory for Space Bioscience & Biotechnology Northwestern Polytechnical University Xi'an China; ^4^ Department of Nuclear Medicine, Xi'an Central Hospital Xi'an Jiaotong University Xi'an China; ^5^ Department of Neurobiology, School of Basic Medicine Fourth Military Medical University Xi'an China; ^6^ Department of Endocrinology, Xi'an Central Hospital Xi'an Jiaotong University Xi'an China; ^7^ Shaanxi Lon‐EV Biotechnology Limited Company Xi'an China; ^8^ Neurology Hospital, Xi'an People's Hospital Northwest University Xi'an China; ^9^ Institute for Regenerative Medicine Texas A&M University School of Medicine College Station Texas USA; ^10^ Graduate College of Biomedical Sciences Western University of Health Sciences Pomona California USA

**Keywords:** cross‐organ, extracellular vesicles, NAFLD, neurovascular complications, PDGFB, type 2 diabetes mellitus

## Abstract

Type 2 diabetes mellitus (T2DM) combined with non‐alcoholic fatty liver disease (NAFLD) exacerbates metabolic dysregulation and neurovascular complications, presenting significant therapeutic challenges. We demonstrate, using SPECT/CT imaging, that extracellular vesicles (EVs) from mesenchymal stromal cells (MSCs) predominantly accumulate in the liver, where they deliver miR‐31‐5p to suppress platelet‐derived growth factor B (PDGFB) produced by hepatic macrophages. This intervention impedes NAFLD progression and establishes a mechanistic link between liver repair and neurovascular improvement. Specifically, single‐nucleus RNA sequencing reveals that PDGFB suppression enhances hippocampal pericyte recovery via the PDGFB‐PDGFRβ axis and orchestrates the activation of growth differentiation factor 11 (GDF11), thus promoting neuroplasticity. Furthermore, AAV injections indicate that hepatic PDGFB modulation recalibrates transthyretin (TTR) dynamics, thereby restoring its neuroprotective functions and preventing its pathological deposition in the brain. These findings position MSC‐EVs as a transformative therapeutic platform that leverages the liver‐brain axis to address the intertwined metabolic and neurovascular complications of T2DM, offering a promising avenue for clinical translation.

## Graphical abstract

Therapeutic extracellular vesicles (EVs) predominantly accumulate in the diabetic liver, delivering miR‐31‐5p to suppress macrophage platelet‐derived growth factor B (PDGFB), thereby impeding non‐alcoholic fatty liver disease (NAFLD) progression and enhancing neurovascular health by promoting pericyte recovery and transthyretin (TTR)‐mediated hippocampal homeostasis through cross‐organ (liver‐brain axis) repair.

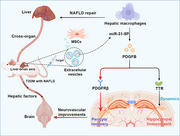

## 1 Introduction

Type 2 diabetes mellitus (T2DM) is a major global health concern, affecting over 400 million individuals worldwide and significantly increasing the risk of multi‐organ complications, including cardiovascular and neurological complications (Crawford and Laiteerapong 2024; Kim et al. 2024). Among its comorbidities, non‐alcoholic fatty liver disease (NAFLD) stands out due to its high prevalence, affecting up to 50% of individuals with T2DM. NAFLD comprises a spectrum of liver pathologies, ranging from benign steatosis to non‐alcoholic steatohepatitis (NASH), a leading cause of cirrhosis and hepatocellular carcinoma (Loomba et al. 2021). Beyond its hepatic manifestations, NAFLD disrupts systemic homeostasis and exacerbates cerebrovascular dysfunction, contributing to blood‐brain barrier (BBB) instability, neuroinflammation, and cognitive decline (Kim et al. 2024; Lu et al. 2024a). Pericytes, essential mural cells, maintain BBB integrity, regulate vascular remodelling and support neurovascular integrity (Sweeney et al. 2016). Accordingly, restoring pericyte function is therefore a promising therapeutic strategy to enhance neurovascular health. These intertwined pathologies highlight the urgent need for therapies targeting both hepatic and neurological complications in T2DM.

Recent advances in extracellular vesicle (EV) research have opened new avenues for treating complex, multi‐organ diseases (Fusco et al. 2024; Carney et al. 2025). EVs, including exosomes and microvesicles, are nanoscale particles that mediate intercellular communication and play critical roles in tissue repair, immune modulation and organ‐specific targeting (Carney et al. 2025; Kang et al. 2021). Mesenchymal stromal cell (MSC)‐derived EVs, in particular, inherit the regenerative and immunomodulatory properties of their parent cells (Chen et al. 2021; Shi et al. 2018) and have shown therapeutic properties in hepatic and neurological disorders (Fusco et al. 2024; Psaraki et al. 2022). However, their ability to address complex, interconnected pathologies across multiple organ systems remains underexplored, particularly in the context of T2DM with NAFLD.

Here, we systematically investigated the therapeutic potential of MSC‐derived EVs in T2DM with NAFLD. Our findings show that EVs preferentially accumulate in the liver, where they suppress platelet‐derived growth factor B (PDGFB) via miR‐31‐5p, effectively impeding NAFLD progression by reducing fibrosis, inflammation and steatosis. Beyond hepatic repair, EV‐induced PDGFB reduction modulates the PDGFB‐PDGFRβ signalling axis and transthyretin (TTR) dynamics, promoting brain pericyte recovery and neurovascular improvements. By leveraging the liver‐brain axis, this study establishes a foundation for developing cross‐organ therapies to address the intertwined metabolic and neurological complications of T2DM.

## 2 Materials and Methods

Detailed methods are provided in the Supporting Information.

### 2.1 Reagents, Primers and Sequences

Shown in Table 1.

**TABLE 1:** Regents, primers, sequences, and other sources.

Regents	Source	Identifier
**Antibodies**		
**CD81**	Affinity Biosciences	AB_2839530
**CD63**	Cell Signalling	AB_2924771
**CD9**	Abcam	AB_2922400
**TSG101**	Abcam	AB_10974262
**Calnexin 43**	Abcam	AB_2069006
**S100β**	Thermo Fisher Scientific	AB_27365
**AQP4**	Thermo Fisher Scientific	AB_26381
**HMGB1**	Proteintech	AB_22329
**PDGFRβ**	Thermo Fisher Scientific	AB_3093309
**P‐PDGFRβ**	Thermo Fisher Scientific	AB_27929
**RGS5**	Proteintech	AB_10644290
**pSMAD2**	Affinity Biosciences	AB_2834844
**SMAD2**	Affinity Biosciences	AB_2835272
**COL1a1**	Abcam	AB_29275
**GDF11**	Affinity Biosciences	AB_28416
**GAPDH**	Affinity Biosciences	AB_2839421
**β‐actin**	Proteintech	AB_2687938
**TTR**	Merck Millipore	AB_10646
**α‐SMA**	Abcam	AB_262054
**VE Cadherin**	Abcam	AB_33168
**iNOS**	Proteintech	AB_2782960
**p‐AKT**	Proteintech	AB_2881201
**AKT**	Proteintech	AB_10912803
**Occludin**	Proteintech	AB_2880820
**Connexin 43**	Proteintech	AB_2880711
**p‐NF‐kappaB (p‐p65)**	Cell Signaling Technology	AB_331284
**NF‐kappaB (p65)**	Cell Signaling Technology	AB_10859369
**MAP2**	Abcam	AB_28953
**PDGFB**	Affinity Biosciences	AB_2833415
**PDGFRβ**	Santa Cruz	AB_10990921
**CD34**	Abcam	AB_3675873
**GDF11**	Affinity Biosciences	AB_2841629
**Goat Anti‐Rabbit IgG H&L (Cy3)**	Abcam	AB_955021
**Goat Anti‐Mouse IgG H&L (Cy3)**	Abcam	AB_10680176
**Goat Anti‐Mouse IgG H&L (FITC)**	Abcam	AB_955241
**Goat Anti‐Rabbit IgG H&L (FITC)**	Abcam	AB_955238
**CD68**	Proteintech	AB_2881049
**α‐SMA**	Abcam	AB_262054
**PDGFB**	Bioss	AB_11085025
**COL1a1**	Abcam	AB_2927551
**F4/80**	BioLegend	AB_893502
**CD11b**	BioLegend	AB_755986
**CD206**	BioLegend	AB_10900231
**CD86**	BioLegend	AB_313159
**Chemicals, peptides, and recombinant proteins**		
**MCD**	ReadyDietech	A06071302
**HFD**	ReadyDietech	D12492
**STZ**	Solarbio	S8050
**RPMI‐1640**	Thermo Fisher Scientific	11875093
**α‐MEM**	Thermo Fisher Scientific	12561056
**FBS**	Sigma–Aldrich	F2442
**M‐CSF**	PeproTech	315‐02
**palmitic acid**	Kunchuang Biotechnology	KC006
**Neurobasal medium**	Thermo Fisher Scientific	21103049
**B27 Supplement**	Thermo Fisher Scientific	17504044
**Penicillin‐Streptomycin**	Novnature	NP2002
**L‐Glutamine**	Thermo Fisher Scientific	25030081
**GDF11 recombinant protein**	PeproTech	120‐11‐20UG
**Luspatercept**	MedChemExpress (MCE)	HY‐P99720
**Dulbecco's modified Eagle medium**	Thermo Fisher Scientific	11965118
**Dulbecco's modified Eagle medium**	Thermo Fisher Scientific	11966025
**Lipopolysaccharide, LPS**	Sigma–Aldrich	L2880
**PDGFB recombinant protein**	MedChemExpress (MCE)	HY‐P7005
**phosphate‐buffered saline, PBS**	Gibco	10010023
**Tin (II) chloride dihydrate**	Sigma–Aldrich	243523
**Technetium‐99m (99mTc)**	Guangdong CI Medicine Co. Ltd.	
**Acetone solution**	Thermo Fisher Scientific	A16S‐4
**Tyrphostin AG1296**	MedChemExpress (MCE)	HY‐13894
**PKH26**	Sigma–Aldrich	MINI26
**CM‐A594**	Invitrogen	A10256
**isoflurane**	Sigma–Aldrich	1349003
**ethanol**	Sigma–Aldrich	65350‐M
**Oil Red O**	Abcam	ab146295
**Sirius Red**	MedChemExpress (MCE)	HY‐D0333
**3% Glutaraldehyde**	Sigma–Aldrich	G5882
**methylene blue**	Sigma–Aldrich	M9140
**uranyl acetate**	Sigma–Aldrich	U18800
**Lead Citrate**	Sigma–Aldrich	L8750
**Nuclei EZ Lysis Buffer**	Sigma–Aldrich	NUC101
**Debris removal solution**	Miltenyi Biotec	130‐109‐398
**proteinase K**	Sigma–Aldrich	P2308
**saline‐sodium citrate (SSC) buffers**	Thermo Fisher Scientific	AM9770
**4',6‐diamidino‐2‐phenylindole (DAPI)**	Thermo Fisher Scientific	R37606
**Radioimmunoprecipitation assay (RIPA) buffer**	Beyotime	P0013B
**Sodium dodecyl sulphate‐polyacrylamide gel electrophoresis (SDS‐PAGE)**	Beyotime	P0012A
**Polyvinylidene fluoride**	Merck Millipore	331789
**2‐Methyl‐2‐pentenoic acid**	Sigma–Aldrich	M1076
**Golgi–Cox solution**	5% potassium chromate, 5% potassium dichromate, and 5% mercuric chloride	
**High‐sucrose solution**	in mmol/L: 2.5 KCl, 1.25 NaH2PO4, 2 Na2HPO4, 2 MgSO4, 213 sucrose, 26 NaHCO3	
**Artificial cerebrospinal fluid**	in mmol/L: 126 NaCl, 2.5 KCl, 1.25 NaH2PO4, 1.25 Na2HPO4, 2 MgSO4, 10 glucose, 26 NaHCO3, 2 CaCl2	
**Internal solution**	in mmol/L: 135 K‐gluconate, 4 KCl, 10 HEPES, 10 sodium phospho‐ creatine, 4 Mg‐ATP, 0.3 Na3‐GTP, and 0.5 biocytin; pH7.2; 265 mOsm	
**Critical commercial assays**		
**TNF‐α ELISA Kit**	R&D SYSTEMS	DTA00D
**iNOS ELISA Kit**	Abcam	ab253217
**IL‐4 ELISA Kit**	R&D SYSTEMS	D4050
**IL‐10 ELISA Kit**	R&D SYSTEMS	D1000B
**VECAM‐1 ELISA Kit**	Abcam	ab201278
**ET‐1 ELISA Kit**	Signalway Antibody	EK1835
**IL‐1β ELISA Kit**	R&D SYSTEMS	MLB00C
**TNF‐α ELISA Kit**	R&D SYSTEMS	MTA00B
**NSE ELISA Kit**	Abcam	ab233626
**BDNF ELISA Kit**	R&D SYSTEMS	DY248
**TTR ELISA Kit**	Signalway Antibody	EK5976
**ATRA ELISA Kit**	JINGMEI BIOTECHNOLOGY	JM‐061654O1
**FT4 ELISA Kit**	FineTest	EM1037
**GDF11 ELISA Kit**	Signalway Antibody	EK14196
**PDGFB ELISA Kit**	R&D SYSTEMS	MBB00
**TGF‐β ELISA Kit**	R&D SYSTEMS	DB100C
**IL‐10 ELISA Kit**	R&D SYSTEMS	M1000B
**IL‐1β ELISA Kit** **Glu ELISA Kit** **GHbA1C ELISA Kit** **Insulin ELISA Kit** **TG ELISA Kit** **LDL‐C ELISA Kit**	R&D SYSTEMS Bioswamp Bioswamp Bioswamp Bioswamp Bioswamp	MLB00C BTK013 MU30349 MU30432 BTK065 BTK012
**ALT ELISA Kit**	JingNing Bio	JN20465
**AST ELISA Kit**	JingNing Bio	JN20681
**QIAamp DNA Micro Kit (50)**	Qiagen	56304
**QIAseq ultralow input library kit**	Qiagen	180492
**Qiagen serum/Plasma Kit**	Qiagen	217184
**QIAseq miRNA library kit**	Qiagen	331505
**High sensitivity DNA Kit**	Agilent	5067‐4626
**Haematoxylin–Eosin staining kit**	MedChemExpress (MCE)	HY‐K0315
**BCA protein assay Kit**	Beyotime	P0012
**RNA easy fast tissue/cell kit**	Qiagen	959035
**High‐capacity cDNA reverse transcription kit**	Qiagen	205411
**Annexin V‐FITC/PI apoptosis kit**	Elabscience	E‐CK‐A211
**Deposited data**		
**Metagenomic next‐generation sequencing**	This paper	PRJNA1181240https://www.ncbi.nlm.nih.gov/sra/
**EV‐miRNA Microarray**	This paper	PRJNA1181238https://www.ncbi.nlm.nih.gov/sra/
**SnRNA‐Seq analysis**	This paper	PRJNA1180752https://www.ncbi.nlm.nih.gov/sra/
**Oligonucleotides**		
**Ctla2a (5’‐3’)**	Forward	CTCCACCCCCTGATCCAAGT
	Reverse	ACACGAGTCTTCTGTGTCTTTCT
**AAV sequences**		
**AAV8‐GP‐1‐PDGFB**	GCCTGCAAGTGTGAGACAGTA	
**AAV8‐GP‐1‐SJ‐ negative control (NC)**	UUCUCCGAACGUGUCACGUTT	
**AAV8‐GP‐13N‐NC**	ACGUGACACGUUCGGAGAATT	
**AAV8‐GP‐PDGFB**	ATGAATCGCTGCTGGGCGCTCTTCCTTCCTCTCTGCTGCTACCTGCGTCTGGTCAGCGCCGAGGGGGATC CCATTCCTGAGGAACTGTATGAAATGCTGAGCGACCACTCCATCCGCTCCTTTGATGATCTTCAGCGCCT GCTGCACAGAGACTCCGTAGATGAAGATGGGGCTGAGCTGGACTTGAACATGACCCGAGCACACTCCGGA GTCGAGTTGGAAAGCTCATCTCGAGGGAGGAGGAGCCTAGGGTCCCTGGCAGCAGCAGAGCCTGCTGTAA TCGCCGAGTGCAAGACGCGCACAGAGGTGTTCCAGATCTCTCGGAACCTCATCGATCGCACCAACGCCAA CTTCCTGGTGTGGCCGCCCTGTGTGGAGGTGCAGCGCTGCTCCGGCTGCTGCAATAACCGCAATGTGCAA TGCCGGGCCTCGCAGGTGCAGATGCGGCCGGTCCAGGTGAGAAAGATTGAGATTGTGCGAAAGAAGCCCA TCTTCAAGAAGGCCACAGTGACCTTGGAGGACCACCTCGCCTGCAAGTGTGAGACAGTAGTGACCCCTCG GCCTGTGACTAGAAGTCCTGGGACATCCAGGGAGCAGCGAGCCAAGACGCCTCAAGCTCGGGTGACCATT CGGACGGTGAGAATCCGCCGGCCCCCCAAAGGCAAGCACCGAAAGTTTAAGCACACCCATGACAAGGCGG CCCTGAAGGAGACCCTCGGAGCCTAG	
**RNA sequences**		
**PDGFB probe**	GCTTCTTTCGCACAATCTCAATCTTTCT‐CY5	
**TTR probe**	CAGCTTCAGACACAAATACCAGTCCAGC‐CY3	
**AgomiR‐31‐5p**	AGGCAAGAUGCUGGCAUAGCUG	
**AgomiR‐24‐3p**	UGGCUCAGUUCAGCAGGAACAG	
**hsa‐mir‐31‐5p mimics**	AGGCAAGAUGCUGGCAUAGCU CUAUGCCAGCAUCUUGCCUUU	
**hsa‐miR‐31‐5p inhibitor**	AGCUAUGCCAGCAUCUUGCCU	
Software and algorithms		
**Scalable nucleotide alignment program**	N/A	https://github.com/amplab/snap
**Fastx toolKit (v0.0.13)**	N/A	http://hannonlab.cshl.edu/fastx_toolKit/
**miRanda**	N/A	http://www.microrna.org/
**Q.Volumetrix MI**	N/A	General Electric Co
**ImageJ Pro Plus V6.0**	N/A	https://imagej.nih.gov/ij/
**SMART v3.0**	N/A	https://www.harvardapparatus.com/panlab‐smart‐video‐tracking
**The cell ranger analysis pipeline (v6.0.2)**	N/A	https://support.10xgenomics.com/single‐cell‐gene‐expression/software/downloads/6.0
**Scanpy (v1.8)**	N/A	https://scanpy.readthedocs.io/
**The cell ranger analysis pipeline (v6.0.2)**	N/A	https://support.10xgenomics.com/single‐cell‐gene‐expression/software/downloads/latest
**Clampfit 10.6**	N/A	https://www.moleculardevices.com/
**MiniAnal software**	N/A	https://www.moleculardevices.com/
**ImageJ Pro Plus V6.0**	N/A	https://imaris.oxinst.com/
**Image Lab**	N/A	https://www.bio‐rad.com/
**Bio‐Rad CFX Manager Software 2.1**	N/A	https://www.bio‐rad.com/
**GraphPad Prism 10.0**	N/A	https://www.graphpad.com/updates/prism‐10‐2‐0‐release‐notes
**SoftMax Pro software**	N/A	https://www.moleculardevices.com/

### 2.2 Ethics Statement

Human umbilical cord tissues were obtained from healthy full‐term mothers with informed consent. All procedures were approved by the Ethics Committee of Xi'an Central Hospital, Xi'an Jiaotong University (Approval No. LAS‐L‐2022‐004‐01), and complied with the standards of the National Health Research Institute. The experimental procedures were performed in accordance with the Guide for the Care and Use of Laboratory Animals (8th edition, 2011) and approved by the ethical committees of Xi'an Jiaotong University Health Science Centre (Approval No. 2021‐1104).

### 2.3 Animals

C57BL/6 mice were purchased from the Experimental Animal Centre of Xi'an Jiaotong University. Male db/db mice, wild‐type (WT) mice, spontaneously hypertensive rats (SHR) and Wistar rats were purchased from Charles River Laboratories (MA, USA). C57BL/6 mice received a methionine‐ and choline‐deficient (MCD) diet to induce NASH (Zhang et al. 2019), while others were placed on a high‐fat diet (HFD) and received streptozotocin (STZ, 60 mg/kg) to induce T2DM (Kojima et al. 2003).

### 2.4 Primary Culture of Cells

MSCs were obtained from Wharton's jelly and cultured in a complete culture medium (CCM), as previously described (Xian et al. 2019; Wang et al. 2020). Considering their essential role in NAFLD progression (Kazankov et al. 2019), bone marrow‐derived macrophages (BMDMs) were collected from the bone marrow of femurs and tibias and cultured with conditioned medium according to previous protocol (Ying et al. 2013). To induce NASH cell model, BMDMs were stimulated with palmitic acid (PA) (Ni et al. 2022). Hippocampal neurons were isolated from mouse embryos and cultured with neurobasal medium (Luo et al. 2021). For the growth differentiation factor 11 (GDF11) intervention, recombinant protein was added to the medium, with or without Luspatercept (ACE‐536, a Smad2/3 inhibitor) administration (Moigneu et al. 2023; Suragani et al. 2014).

### 2.5 Cell Lines

Human BMDMs, brain microvascular pericytes, embryonic kidney 293 (HEK293) cells, and RAW 264.7 cells were obtained from ScienCell Research Laboratories. An immune injury cell model was induced by treating hBMDMs with lipopolysaccharide (LPS) (Zhao et al. 2024). Varying concentrations of PDGFB recombinant protein (10, 50, 100 ng/mL) were added to the culture medium of pericytes as previous reports (Gu et al. 2021). Recombinant PDGFB protein and/or AG1296 (a PDGFR tyrosine kinase inhibitor) were employed to elucidate PDGFB‐PDGFRβ signalling in pericytes (Gu et al. 2021).

### 2.6 EV Isolation

The third to fifth passage of MSCs were cultured with CCM containing 10% EV‐depleted foetal bovine serum (FBS) (Kornilov et al. 2018). Afterward, cell supernatants were collected and underwent a series of centrifugation steps using a ST16R centrifuge (Thermo Fisher Scientific, USA) (Zhao et al. 2022; Thery et al. 2006). These supernatants were then further centrifuged at 100,000×*g* for 90 min with an XPN‐100 ultracentrifuge (Beckman Coulter, USA) to obtain the EV pellets. The collected pellets were resuspended in sterile phosphate‐buffered saline (PBS) and subjected to an additional centrifugation at 120,000×*g* for 70 min to eliminate free proteins and impurities. Also, crude EVs were isolated by centrifuging the cell supernatant at 100,000×*g* for 1.5 h. The pellet was resuspended and loaded onto a sucrose gradient (10%–82%, 1.002–1.34 g/mL density) for centrifugation at 100,000×*g* for 16 h. Six density fractions were collected, diluted in PBS, and centrifuged again at 100,000×*g* for 1.5 h to pellet purified EVs (P‐EVs) (Crewe et al. 2018). These isolated EVs were stored at −80°C until further use.

### 2.7 Quality Control of MSC‐EVs

Quality control of EVs was performed using metagenomic next‐generation sequencing (mNGS) by Hugobiotech Co., Ltd. (Beijing, China), as previously documented (Lu et al. 2024b). This database included over 20,000 microbial genomes sourced from NCBI, comprising 11,910 bacterial, 7103 viral, 1046 fungal, and 305 parasitic genomes. Finally, the microbial compositions of the samples were determined and analysed using mNGS. The microbial data were deposited in the NIH National Library of Medicine (accession number: PRJNA1181240).

### 2.8 EV Characterization and Bioactivity Test

Three EV preparations passed quality control (Figure S1A), and purified EVs (P‐EVs) were characterized using Western blotting to detect representative markers. These markers included CD81, CD63, CD9, and TSG101 as positive indicators, and Calnexin (Table 1) as a negative control to confirm the absence of cellular contaminants (Witwer et al. 2019). The morphology and size distribution of the EVs were analysed using transmission electron microscopy (TEM) and nanoparticle tracking analysis (NTA). To evaluate the immunomodulatory activity of these EVs, hBMDMs were pretreated with 10 µg/mL EVs or P‐EVs for 12 h to ensure uptake (Zhao et al. 2022), followed by stimulation with LPS (Zhao et al. 2024). The cell supernatants were collected and analysed using ELISA kits for TNF‐α, iNOS, IL‐4 and IL‐10 (Table 1), following the manufacturer's instructions.

### 2.9 EV Labelling

For in vivo tracking, EVs were incubated with 0.01% tin (II) chloride dihydrate for 5 min, then labelled with various doses of 99mTcO_4⁻_ at room temperature for 30 min. After purification with Sephadex G‐25 columns, labelling efficiency was measured by instant thin‐layer chromatography‐silica gel (ITLC‐SG) and a radioactive scanner. CCK8 assays showed no effect on EV function. Stability was assessed by radiochemical purity over 24 h. Finally, 148 MBq 99mTcO_4⁻_ was used to label 100 µg EVs (50 µg/150 µL, 74 MBq ^99m^Tc) (Chung et al. 2024; Yang et al. 2024). For ex vivo tracking, the vesicles were labelled with PKH26 and indicated using EV marker CD9, following established protocols (Long et al. 2017; Lu et al. 2025).

### 2.10 EV‐miRNA Analysis via NGS

EV RNA was extracted using the Qiagen Serum/Plasma Kit and evaluated with an Agilent 2100 BioAnalyzer. NGS libraries were prepared with the QIAseq miRNA Library Kit, and sequencing was performed on an Illumina NextSeq 6000 platform. Clean reads were mapped to the GRCh38 genome, and miRNAs were identified using miRBase and miRCat. Differentially expressed miRNAs (FDR < 0.05, |log2FC| > 1) were analysed with edgeR. Target genes were predicted with miRanda, and KEGG analyses were conducted for functional insights. The miRNA data were deposited in the NIH National Library of Medicine (accession number: PRJNA1181238).

### 2.11 EV Administration

EV batches were randomly selected, ensuring that each biological replicate included EVs derived from all three preparations to account for potential variability among batches. MSC‐EVs were administered at a dose of 2 mg/kg of body weight (equating to approximately 10^12^/kg) (Xian et al. 2019; Cheng and Kalluri 2023). The intervention time points for db/db (20 weeks old) and MCD (12 weeks old) mice were primarily chosen to enhance the reversal effect of EVs on MAFLD (Yang et al. 2020; Zou et al. 2024). To track the EVs in vivo, db/db, MCD, HFD/STZ, SHR, or age‐matched control mice / rats were administered EVs. An equivalent dose of free technetium (^99m^TcO4‐) was injected as a control in the tracking experiments.

### 2.12 SPECT/CT Imaging

SPECT/CT imaging was performed to evaluate the biodistribution of EVs in vivo, as previously described (Yang et al. 2024). Each group underwent a 1200‐s SPECT planar scan. The SPECT/CT imaging used a 256 × 256 acquisition matrix with a 1.5 zoom factor, capturing each tomographic angle for 20 s and CT scans with a 1 mm sectional thickness. Gamma camera (Mediso nanoScan, Hungary) images were taken at 1‐, 6‐, 24‐, and 48‐h post‐injection.

### 2.13 Pharmacokinetics Analysis of Infused EVs

Pharmacokinetic analysis was performed to evaluate the temporal distribution of ^99m^Tc‐labeled EVs in vivo. Radiation measurements using a water model that mimicked the volume and total dose administration in rodents (Uhl et al. 2015). Subsequent evaluations in live rodents incorporated both physical (1/T) and biological half‐lives (1/Tb). The systemic drug residue percentage was calculated using the formula: Systemic drug residue (%) = [e^(−0.693/Te)] × 100.

### 2.14 AAV Production and Injection

Recombinant liver‐targeted AAV8‐GP‐1‐PDGFB, AAV8‐GP‐1‐SJ‐negative control (NC), AAV8‐GP‐PDGFB and AAV8‐GP‐13N‐NC, were synthesized by GenePharma (Shanghai, China) (Table 1). The db/db mice received tail vein injections of either AAV8‐GP‐1‐PDGFB (db/db‐PDGFB^AAV−^) or AAV8‐GP‐1‐SJ‐NC (db/db‐NC) to downregulate PDGFB in the liver. WT mice were injected via the tail vein with either AAV8‐GP‐PDGFB (WT‐PDGFB^AAV+^) or AAV8‐GP‐13N‐NC (WT‐NC) to upregulate PDGFB in the liver.

### 2.15 Pathological Staining

Liver tissues from the experimental mice were prepared and stained with H&E, Sirius red, and Oil Red O, as manufacturer's instructions. NASH cell models were also subjected to Oil Red O staining to assess lipid accumulation. Images were captured using a microscope. For statistical accuracy, three random fields were selected and imaged at 200× magnification from each sample in every group. Image analysis was performed using ImageJ Pro Plus V6.0 (Bethesda, Maryland, USA).

### 2.16 Neurobehavioral Tests

Neurobehavioral tests, including the Y‐maze, Open Field Test (OFT), and Elevated Plus Maze (EPM), were performed on db/db mice at 2‐, 4‐, and 6‐ weeks post‐infusion, MCD mice at 2‐ and 4‐weeks post‐treatment, either db/db‐PDGFB^AAV−^ or db/db‐NC groups, and age‐matched vehicle control (Liu et al. 2021; Zhang et al. 2021). Behaviour was analysed using SMART v3.0 software (Panlab, USA), measuring parameters such as alternation triplet, speed, time spent in central area, and entries in to open arms. Alternation triplet in Y maze was calculated as: % Alternation = (Number of Alternations/[Total number of Arm Entries – 2]) × 100.

### 2.17 Transmission Electron Microscope (TEM)

The brain hippocampi were isolated and processed as previously described (Yang et al. 2024). Hippocampal sections were stained with methylene blue, while ultrathin sections were cut with a diamond knife and stained with uranyl acetate and lead citrate. Sections were examined using a transmission electron microscope.

### 2.18 SnRNA‐Seq Analysis

Hippocampi from db/db mice 4 weeks post‐injection with EV or PBS, and WT controls (*n* = 3; samples pooled from two mice per group), were processed for single‐nucleus RNA sequencing (snRNA‐seq) using the 10× Genomics platform (∼6000 nuclei/sample) and sequenced on Illumina NovaSeq 6000. In total, 53,307 single‐cell transcriptomes were analysed. Library size normalization followed established protocols (Wolf et al. 2018, 2019). Comparative clustering assessed cell population shifts between groups, with Cluster 24 further subclustered for enhanced resolution. Pseudotime analysis elucidated lineage trajectories among sub‐clusters 0–2. Marker genes were identified using Seurat's FindAllMarkers (Wilcoxon test, adjusted *p* < 0.01, |fold change| > 2). Functional enrichment (GO, HALLMARK, Reactome) was performed via g:Profiler2. Cell–cell interactions were analysed with CellPhoneDB, annotating ligand/receptor pairs and assessing significance (*p* < 0.05). All sequencing and bioinformatics were conducted by Genechem Co., Ltd. (Shanghai, China). Data are available at NCBI (PRJNA1180752).

### 2.19 Golgi–Cox Staining

Golgi–Cox staining was performed to assess changes in the hippocampal synaptic structure as previously described (Zhang et al. 2020; Wang et al. 2021). 9–10 pyramidal neurons were randomly selected from the experimental groups (Guo et al. 2019). A three‐dimensional analysis of dendritic morphology was conducted using a computerized tracing system. The summary analysis of the neuronal data, including total dendritic length, number of dendritic branch points, total dendritic volume, and dendritic complexity index was collected using Neurolucida Explorer (MBF Bioscience).

### 2.20 Whole‐Cell Patch‐Clamp Recording

Whole‐cell patch‐clamp recording was performed 4 weeks after administration, as previously established protocol (Guo et al. 2018; Wang et al. 2019a). Coronal slices were prepared using a vibrating oscillator and placed in a recording tank and continued infusion of artificial cerebrospinal fluid (Table 1). Recording micropipettes were pulled in a horizontal pipette puller with a tip resistance of 3–6 MΩ. Patch pipettes were filled with the internal solution (Table 1). Currents recordings were made using an Axopatch 700B amplifier (Axon Instruments). Signals were filtered at 20 kHz and sampled at 5 kHz with a Digidata 1550B and Clampex 10.7 (Molecular Devices). The data were stored on a computer and analysed using Clampfit 10.6 and MiniAnal software (Molecular Devices, CA, USA).

### 2.21 Fluorescence In Situ Hybridization (FISH)

FISH was employed to detect specific genetic markers in liver and brain specimens from experimental groups 4 weeks following EV therapy (Guo et al. 2019). Fluorescently labelled probes targeting PDGFB mRNA in liver macrophages (F4/80) and TTR mRNA in hippocampal endothelial cells (CD34) was employed in this study. Fluorescent signals were captured using a confocal microscope. The spatial distribution and intensity of PDGFB mRNA expression were quantified using ImageJ Pro Plus V 6.0 software (Bethesda, Maryland, USA).

### 2.22 miRNA Modification and Treatment

miR‐31‐5p was selected for its ability to target PDGFB (Table S1). For in vitro experiments, the miRNA inhibitor, mimic, and NC were conjugated with 6‐Carboxyfluorescein (FAM) and synthesized by GenePharma (Shanghai, China). These constructs were introduced into EVs or HEK‐293 cell‐derived EVs following established protocols (Wang et al. 2019b). Primary culture of BMDMs were pre‐treated with 10 µg/mL of these miRNA‐modified EVs, followed by PA stimulation. The successful incorporation of miRNA into EVs was confirmed via immunofluorescence and quantitative PCR (qPCR). For in vivo studies, miR‐31‐5p^agomir^ and agomir NC were synthesized by Ribobio (Guangzhou, China) (Table 1), and administered to MCD mice through tail vein injections (Zou et al. 2024).

### 2.23 Flow Cytometry

MSCs were identified via fluorescence flow cytometry using antibodies against CD105, CD90, CD73, CD45 and CD34 (Table 1) to confirm their mesenchymal origin (Mushahary et al. 2018). BMDMs and RAW 264.7 cells were analysed using antibody panels with F4/80, CD11b, CD206 and CD86 (Table 1) to delineate macrophage subpopulations and assess polarization states (Gordon and Taylor 2005). Apoptosis of pericytes was detected using the Annexin V‐FITC/PI Apoptosis Kitfollowing manufacturer's guidelines. FlowJo software was employed for data analysis, ensuring robust identification and quantification of cellular phenotypes and apoptotic states.

### 2.24 ELISA

Cell supernatant, serum, and tissue samples were harvested from the experimental group at the indicated time points. ELISA kits were employed to quantify the levels of various biomarkers, including tumour necrosis factor‐alpha (TNF‐α), inducible nitric oxide synthase (iNOS), interleukin 4 (IL‐4), interleukin 10 (IL‐10), PDGFB, transthyretin (TTR), free thyroxine (FT4), all‐trans retinoic acid (ATRA), GDF11, transforming growth factor‐beta (TGF‐β), interleukin 1 beta (IL‐1β), brain‐derived neurotrophic factor (BDNF), vascular cell adhesion molecule‐1 (VCAM‐1), endothelin‐1 (ET‐1), neuron‐specific enolase (NSE), Glucose (Glu), Glycated Haemoglobin A1C (GHbA1C), Insulin, low‐density lipoprotein cholesterol (LDL‐C), triglycerides (TG), alanine aminotransferase (ALT), and aspartate aminotransferase (AST) among the experimental groups (Table 1). Absorbance was measured at 450 nm using a FlexStation 3 Microplate Reader, and data were processed using SoftMax Pro software.

### 2.25 Immunofluorescence and Histochemistry

Cell samples and tissue sections were obtained from experimental groups for immunostaining, following established protocols (Xian et al. 2019; Long et al. 2017). The primary antibodies comprised against F4/80, glial fibrillary acidic protein (GFAP), CD9, CD34, PDGFB, GDF11, CD68, alpha‐smooth muscle actin (α‐SMA), neuronal nuclei (NeuN), microtubule‐associated protein 2 (MAP2) and platelet‐derived growth factor receptor‐beta (PDGFRβ) (Table 1), followed by incubation with appropriate secondary antibodies (Table 1). For imaging, an optical microscope or a confocal microscope was used. 3D reconstruction of vascular makers was performed using Imaris9.0 (Bitplane, Switzerland). Data analysis was conducted using ImageJ Pro Plus V6.0 (Bethesda, Maryland, USA).

### 2.26 Western Blotting

Cell and tissue samples were collected from experimental groups and prepared as previously described (Yang et al. 2024). Primary antibodies against PDGFRβ, phosphorylated PDGFRβ (pPDGFRβ), G‐protein signalling 5 (RGS5), GDF11, PDGFB, CD81, CD63, CD9, TSG101, Calnexin, S100β, aquaporin 4 (Aqp4), high mobility group box 1 (HMGB1), phosphorylated small mothers against decapentaplegic 2 (pSMAD2), small mothers against decapentaplegic 2 (SMAD2), Synapsin I (SYN1), p‐protein kinase B (p‐AKT), AKT, p‐nuclear factor kappa‐light‐chain‐enhancer of activated B cells (p‐NF‐κB/p‐p65), p65, VE‐Cadherin, Occludin, and Connexin 43, GAPDH, and β‐actin (Table 1). Membranes were incubated with the appropriate secondary antibodies (Table 1). Each sample was analysed with at least one technical replicate to ensure reliability. Protein bands were visualized using a Bio‐Rad imaging system and quantified with Image Lab software.

### 2.27 qPCR

qPCR was conducted to evaluate the expression of miRNAs (miR‐31‐5p, miR‐24‐3p) and genes (PDGFB, TTR, Ctla2a) in EVs, cells, or tissues as previously described (Zhao et al. 2022; Yang et al. 2024). Total RNA was isolated using the RNA Easy Fast Tissue/Cell Kit. Primer sequences (Table 1) optimized by Takara Bio Inc. (Table 1). qPCR was carried out using the TB Green Premix Ex Taq II on a CFX Connect Real‐Time PCR System. Relative gene or miRNA expression levels were determined using the 2^‐ΔΔCT method, with GAPDH or U6 serving as internal controls. All reactions were performed in triplicate to ensure reproducibility and analysed with Bio‐Rad CFX Manager Software 2.1.

### 2.28 Statistical Analysis

All statistical analyses were conducted using GraphPad Prism 10.0 (GraphPad Software, USA). Data are presented as mean ± standard error of the mean (SEM). Detailed statistical information can be found in the figure legends. For comparisons involving multiple groups, one‐way ANOVA and repeated measures ANOVA were used, followed by Bonferroni post hoc tests to account for potential Type I errors. For comparisons between two groups, an unpaired two‐tailed Student's *t*‐test was utilized for normally distributed data, while the Mann–Whitney *U* test was applied for non‐normally distributed data. Statistical significance was defined as *p* < 0.05.

## 3 Results

### 3.1 EVs Target the Liver to Alleviate NAFLD Changes and Metabolic Dysregulation

**FIGURE 1:**
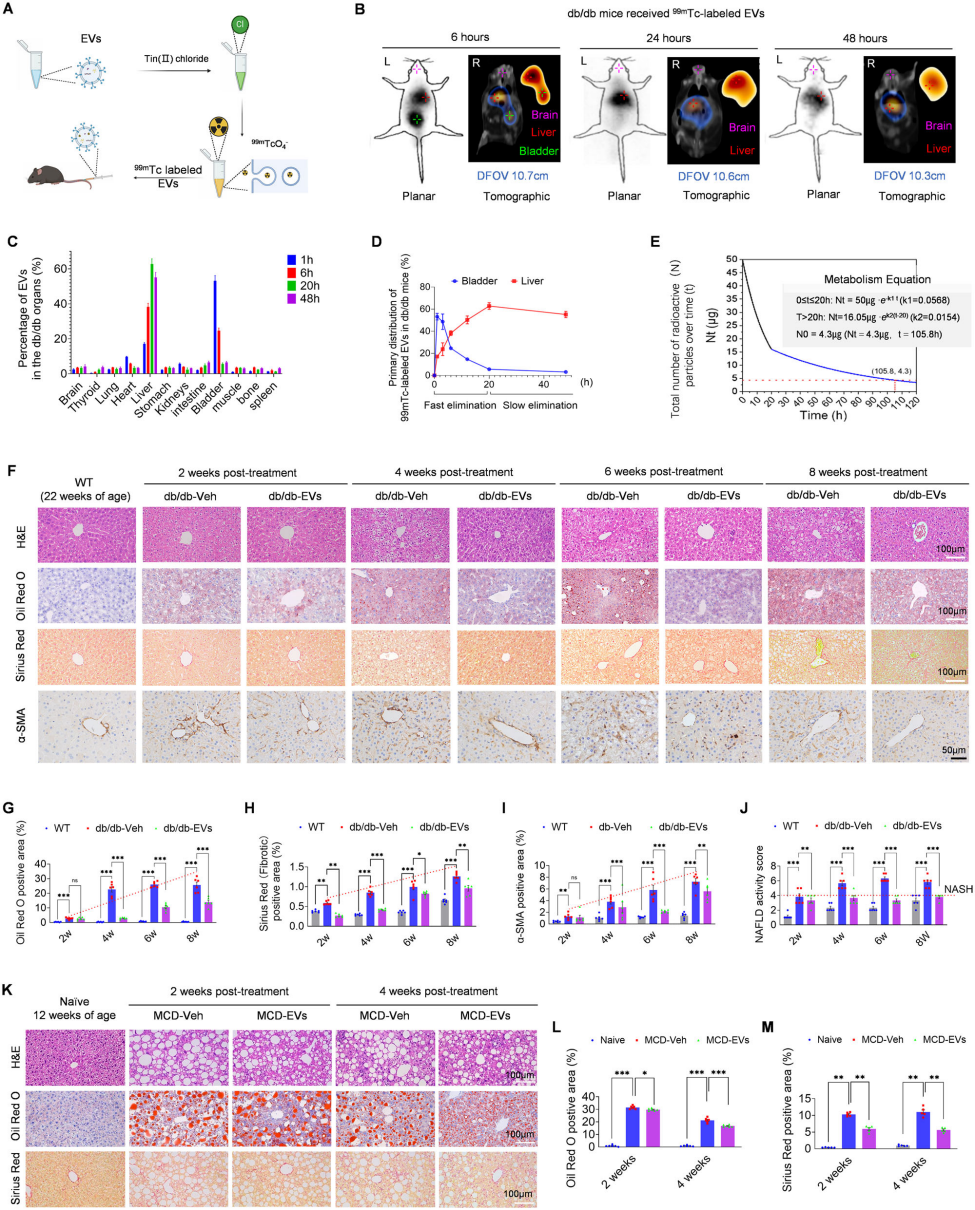
**EV distribution and pathological assessments in NAFLD mice**. (A) Schematic procedures of EVs labelling with ^99m^Tc, created using Biorender.com. (B) SPECT/CT imaging of ^99m^Tc‐labeled EVs in db/db mice at 6‐, 24‐, and 48‐h post‐injection (planar and tomographic views). Images show predominant liver uptake over time. Display fields of view (DFOVs): 10.7, 10.6 and 10.3 cm. (C) Organ biodistribution of EVs at 1‐, 6‐, 24‐, and 48‐h post‐injection, demonstrating predominant liver and bladder accumulation (*n* = 8). (D) EV clearance kinetics from the liver and bladder at different time points, showing distinct fast and slow elimination phases (*n* = 8). (E) Total EV decay modelled using a metabolism equation, illustrating biphasic elimination kinetics (*n* = 8). (F) Representative liver histology in db/db mice at 2‐, 4‐, 6‐, and 8‐weeks post‐treatment. Staining includes H&E (morphology), Oil Red O (lipid deposition), Sirius Red (fibrosis) and α‐SMA (activated hepatic stellate cells, HSCs). WT mice serve as controls. Scale bars: 100 µm (H&E, Oil Red O, Sirius Red) and 50 µm (α‐SMA). (G–I) Quantification of Oil Red O, Sirius Red and α‐SMA staining in db/db mice, showing significant reductions in lipid accumulation, fibrosis, and HSC activation in EV‐treated mice (db/db‐EVs) compared to vehicle‐treated controls (db/db‐Veh) (*n* = 6). (J) NAFLD activity scores, demonstrating attenuated progression to NASH in db/db‐EVs mice compared to db/db‐Veh controls (*n* = 6). (K) Representative liver histology in MCD mice at 2‐ and 4‐weeks post‐treatment. Staining includes H&E, Oil Red O, and Sirius Red, with naïve (healthy) mice as controls. Scale bars: 100 µm. (L, M) Quantification of Oil Red O (*n* = 6) and Sirius Red (*n* = 6) staining in MCD mice, showing significantly reduced lipid deposition and fibrosis in EV‐treated groups (MCD‐EVs) compared to vehicle‐treated controls (MCD‐Veh). All data are presented as mean ± SEM. “*n*” represents biological replicates. Statistical analysis was performed using one‐way ANOVA with Bonferroni post hoc tests. Significance levels: ns (*p* > 0.05); *p < 0.05; **p < 0.01; ***p < 0.001; ****p < 0.0001.

Building on the immunomodulatory activity and therapeutic potential of MSCs derived from human umbilical cords (Chen et al. 2021; Kaur et al. 2024), EVs from three donors were successfully characterized and designated as therapeutic agents (Figure S1A–H). To evaluate their biodistribution, EVs were labelled with ^99m^Tc (Figures 1A and S1I) and administered via tail vein injections in multiple T2DM and/or NAFLD mouse models. SPECT/CT imaging revealed that, compared to ^99m^TcO4‐ tracking (Figure S2A,B), ^99m^Tc‐labeled EVs predominantly accumulated in the liver of db/db mice, a model of T2DM with NAFLD (Figure 1B). Pharmacokinetic analysis over 1–48 h confirmed sustained liver accumulation in db/db (Figure 1C–E,) and WT mice (Figure S2C,D). This was further validated in the HFD‐STZ model, a T2DM model (Figure S3E–H), and MCD, a NASH model representing severe NAFLD (Figure S3I–L) mice. Notably, EVs accumulated primarily in the kidneys of spontaneously hypertensive rats (SHR), a metabolic disease model unrelated to NAFLD (Figure S3M–O). Collectively, these findings demonstrate that infused EVs preferentially target the liver in T2DM with NAFLD.

Given the liver‐trophic properties of EVs, we next evaluated their therapeutic effects in recipient mice. In db/db mice, EV infusion (db/db‐EVs) significantly reduced tissue injury, steatosis, and fibrosis, as evidenced by H&E staining and decreased positive areas for Oil Red O, Sirius Red, and α‐SMA (a marker of activated hepatic stellate cells) staining over 4–8 weeks post‐treatment (Figure 1F–I). EV‐treated db/db mice also exhibited lower NAFLD activity scores compared to vehicle‐treated controls (db/db‐Veh; Figure 1J). Similar pathological improvements were observed in MCD mice, where EV treatment (MCD‐EVs) alleviated steatosis and fibrosis within 2–4 weeks post‐injection (Figure 1K–M). In addition to these histological changes, EV therapy significantly improved metabolic dysregulation in db/db mice compared to vehicle controls after 4 weeks (Figure S3A–G). Remarkably, macroscopic liver repair was evident in EV‐treated db/db mice at the same time point (Figure S3H). Ex vivo EV tracking revealed preferential uptake by liver macrophages and hepatic stellate cells (HSC) (Figure S3I, J), suggesting their potential regulatory role in the management of NAFLD (Hammerich and Tacke 2023). Consistently, EV therapy reduced the expression of fibrosis markers (COL1a1), macrophage markers (CD68), and α‐SMA in both db/db and MCD mice (Figure S3K,L). Together, these findings demonstrate that infused EVs effectively prevent the progression of NAFLD to NASH while alleviating systemic metabolic dysfunction, highlighting a precise therapeutic strategy with significant potential for treating T2DM‐associated NAFLD.

### 3.2 Liver‐Targeted EVs Improve NAFLD‐Associated Neurological Outcomes

**FIGURE 2:**
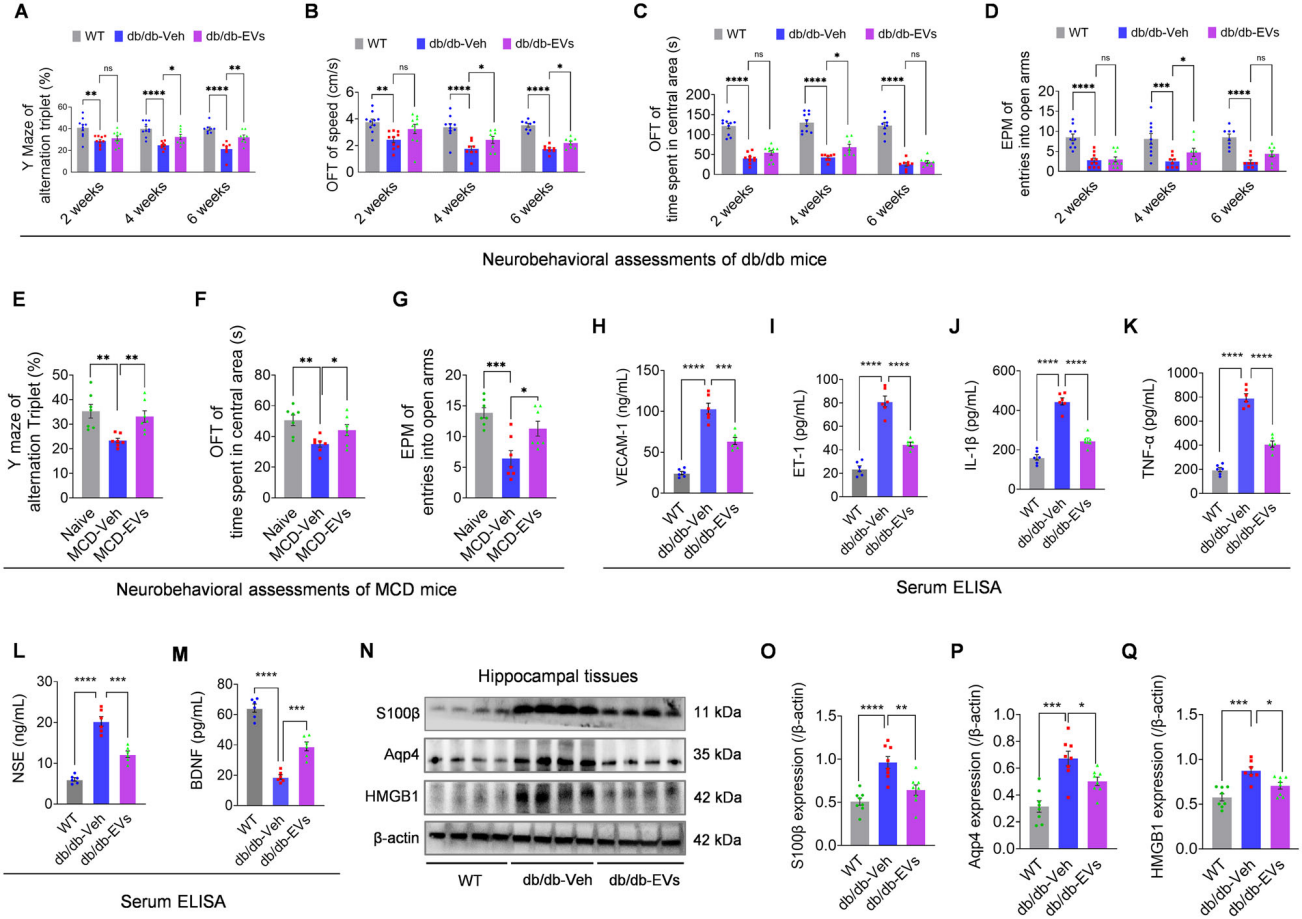
**Assessments of neurological outcomes in NAFLD mice**. (A–D) Neurobehavioral assessments including percentage of alternation triplets in the Y‐maze (A), speed (B) and time spent in the central area (C) in the open field test (OFT), and number of entries into the open arms (D) in the elevated plus maze (EPM) of db/db mice following EV treatment at 2, 4, and 6 weeks. Sample sizes for each time point: 2 weeks (*n* = 10); 4 weeks (WT, *n* = 10; db/db‐Veh and db/db‐EVs, *n* = 8); 6 weeks (*n* = 8). (E–G) Neurobehavioral tests in MCD mice 4 weeks post‐treatment, including the Y maze (A), OFT (B), and EPM (C) (*n* = 8). (H–M) ELISA analysis of serum biomarkers across groups (*n* = 6): VCAM‐1 (H), ET‐1 (I), IL‐1β (J), (H) TNF‐α (K), NSE (L) and BDNF(M). (N–Q) Western blots (N) and quantification of hippocampal neural damage markers for S100β (O), Aqp4 (P), and HMGB1 (Q) in tissues from WT, db/db‐Veh, and db/db‐EVs groups. β‐actin was used as a loading control (*n* = 8). Data are presented as mean ± SEM. “*n*” represents biological replicates. Statistical analysis was performed using one‐way ANOVA with Bonferroni post hoc tests. Significance levels: ns (*p* > 0.05); *p < 0.05; **p < 0.01; ***p < 0.001; ****p < 0.0001.

To assess the systematic effects of liver‐targeted EVs in the context of T2DM and NAFLD (van Sloten et al. 2020; Hadjihambi et al. 2023), we conducted neurobehavioral assessments, including the Y‐maze, OFT, and EPM, at 2‐, 4‐, and 6‐weeks post‐treatment. EV‐treated mice exhibited significant improvements in cognitive and behavioural outcomes, with increased alternation triplets in the Y‐maze (Figure 2A), greater speed and increased time spent in the central area during the OFT (Figures 2B,C), and more open‐arm entries in the EPM (Figure 2D), particularly at 4 weeks post‐treatment. However, no significant neurobehavioral changes were observed at 2 or 6 weeks in db/db mice, likely reflecting the time‐dependent effects of EVs and the advanced pathology of NAFLD at later stages (Figure 1E–I). Similar behavioural improvements were observed in MCD mice at 4 weeks post‐treatment (Figure 2E–G), further supporting the therapeutic potential of EVs on sever NAFLD‐associated brain dysfunction (Hadjihambi et al. 2023). These results suggest that EVs improve neurobehavioral performance in T2DM with NAFLD.

Mechanistically, EV therapy reduced systemic markers of vascular injury (VCAM‐1, ET‐1), inflammation (IL‐1β, TNF‐α), and neuronal damage (NSE), while significantly increasing BDNF, a key neuroprotective molecule, at 4 weeks post‐treatment in db/db mice (Figure 2H–M). Given that these circulating risk factors and neuroprotective molecules are well‐established in diabetic brain pathology (Lenart et al. 2019; Biessels et al. 2020), our findings indicate that EVs provide broad systemic benefits to the db/db brain. Considering the vulnerability of the hippocampus (Johnson 2023; Biessels and Reagan 2015), markers of hippocampal damage, including S100β (neuronal and astrocytic damage), Aqp4 (brain oedema and BBB dysfunction), and HMGB1 (cell damage marker), were significantly reduced in db/db mice following EV infusion (Figure 2N–Q), further supporting the potent neuroprotective effects of EVs in T2DM with NAFLD. In summary, these findings demonstrate that while EVs primarily target the liver, they also improve neurological outcomes in NAFLD‐related conditions, highlighting their potential to mediate cross‐organ repair and address neurological dysfunction in T2DM with NAFLD.

### 3.3 SnRNA‐Seq Reveals Hippocampal Vascular Repair and TTR Modulation

**FIGURE 3:**
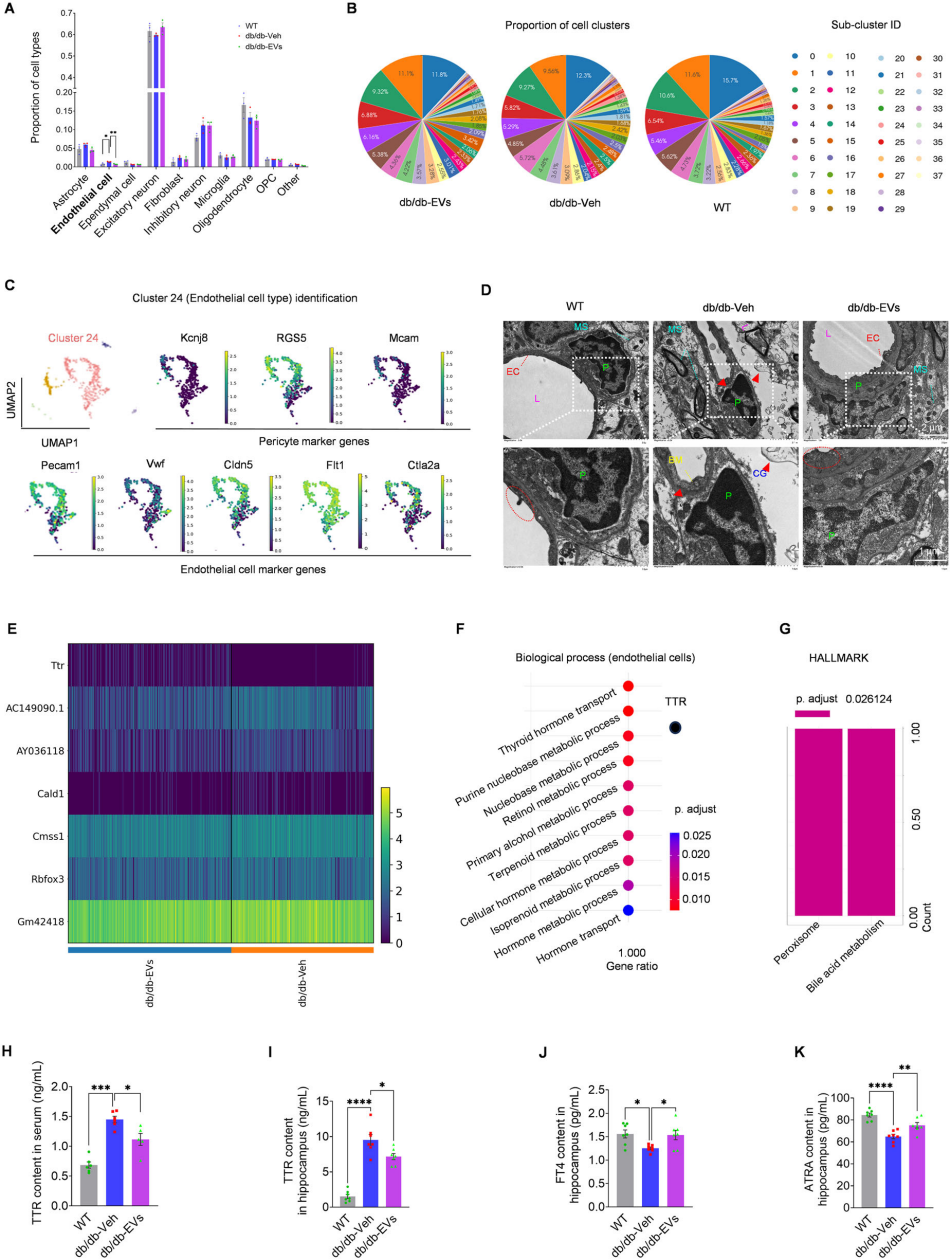
**Impact of EVs on cell populations and TTR changes in db/db hippocampus**. (A) Bar graph illustrating the proportions of major hippocampal cell types across WT, db/db‐Veh, and db/db‐EVs groups 4 weeks post‐treatment (*n* = 3, pooled hippocampi from two mice to generate one biological replicate). (B) Pie charts showing the distribution of hippocampal cell clusters within each group. (C) UMAP plots identifying Cluster 24, characterized as a pericyte and endothelial cell complex. Cluster 24 co‐expresses pericyte markers (Kcnj8, RGS5, Mcam) and endothelial markers (Vwf, Cldn5, Flt1, Ctla2a). (D) Representative TEM images of hippocampal microvasculature showing structural improvements in db/db mice following EV therapy (*n* = 4). Features include pericytes (P), basement membrane (BM), cell gaps (CG), lumen (L), endothelial cells (EC), and myelin sheath (MS). Red dashed boxes highlight tight junctions, and red arrows indicate perivascular oedema. Scale bars: upper images = 2 µm; lower images = 1 µm. (E) Heatmap showing gene expression of TTR and other differentially expressed genes (DEGs) in Cluster 24. TTR was significantly upregulated in the db/db‐EVs group compared to db/db‐Veh (absolute fold change ≥1.2, adjusted *p* ≤ 0.05). (F) Biological process (BP) enrichment (g: Profiler2) associating TTR upregulation with BPs such as thyroid hormone transport and retinol metabolism processes in EV‐treated db/db hippocampus. (G) HALLMARK Gene Set enrichment (g: Profiler2) associating TTR upregulation with pathways such as Bile acid metabolism in db/db hippocampus following EV therapy. (H, I) ELISA of TTR concertation in both serum (H) and hippocampal tissue (I) across WT, db/db‐Veh, and db/db‐EVs groups (*n* = 5–7). (J, K) ELISA results show significantly increased levels of free thyroxine (FT4) (K) and all‐trans retinoic acid (ATRA) (L) in hippocampal tissues of EV‐treated db/db mice, compared to vehicle controls (*n* = 7). Data are presented as mean ± SEM. “*n*” represents biological replicates. Statistical significance was determined by one‐way ANOVA followed by Bonferroni's post hoc test (A, H‐K). Significance levels: *p < 0.05; **p < 0.01; ***p < 0.001; ****p < 0.0001.

To elucidate the mechanisms underlying EV‐induced neurological improvements in db/db mice, we performed single‐nucleus RNA sequencing (snRNA‐Seq) on hippocampal tissues 4 weeks post‐treatment. After stringent quality control and doublet removal, 53,307 nuclei were retained and a median of 3254 genes and 10,174 unique molecular identifiers (UMIs) per nucleus were achieved (Table S1), representing major brain cell types, including excitatory and inhibitory neurons, oligodendrocytes, astrocytes, microglia, oligodendrocyte precursor cells (OPCs), endothelial cells, ependymal cells, fibroblasts and an immune cell population of potential blood origin (Figure S4A,B). Unsupervised clustering identified 38 transcriptionally distinct clusters (Figure S4C; Table S2). Among these, Cluster 24, corresponding to a pericyte‐endothelial cell complex (Figures 3A–C and S4B–D), was significantly restored in EV‐treated mice compared to vehicle controls (Figure 3A; Table S3). Marker gene analysis identified *Ctla2a* as a key marker of Cluster 24 (Figure S4A,E), and its downregulation in EV‐treated mice was confirmed by qPCR (Figure S4F). TEM further demonstrated structural improvements in hippocampal microvasculature following EV therapy (Figure 3D). Together, these findings indicate that EV therapy promotes hippocampal vascular repair in db/db mice.

Cell–cell communication analysis highlighted the functional importance of Cluster 24 in neurovascular repair, showing strong interactions with neurovascular units, including excitatory neurons and astrocytes (Figure S5A). Differentially expressed gene (DEG) analysis revealed that EV therapy induced significant upregulation of *transthyretin (TTR)* in Cluster 24 (Figures 3E and S5B,C) and its interacting cell populations (Figure S5D,E; Table S4). TTR, primarily synthesized by the liver and choroid plexus, plays a critical role in transporting thyroxine (T4) and retinol (vitamin A) (Magalhães et al. 2021). Functional enrichment analysis linked EV‐induced TTR upregulation to neuroprotective processes (Figures 3F,G and S5F,G), such as thyroid hormone transport, retinol metabolism and bile acid metabolism, which are essential for maintaining neurological function (Tian et al. 2024; Kang and Koh 2023; Liu et al. 2024).

Interestingly, ELISA results revealed reduced TTR protein levels in both serum and hippocampal tissues of EV‐treated db/db mice, compared to db/db‐Veh groups (Figure 3H,I), despite the observed upregulation of *TTR* mRNA. This discrepancy suggests an adaptive mechanism involving post‐transcriptional regulation or enhanced protein turnover in the hippocampus following EV therapy. Supporting this hypothesis, EV‐treated mice showed restored levels of free T4 (FT4) and all‐trans retinoic acid (ATRA, a metabolite of retinol) in the hippocampus (Figure 3J,K), both of which are critical for neuronal signalling and brain homeostasis (Tian et al. 2024; Wołoszynowska‐Fraser et al. 2020). Moreover, pathological TTR deposition, which has been implicated in neurovascular damage and cognitive dysfunction (Adams et al. 2023), was mitigated in EV‐treated mice (Figure 3H,I), underscoring the importance of TTR modulation in db/db context. Collectively, these findings reveal that EV therapy promotes hippocampal vascular repair and modulates TTR dynamics to alleviate neurovascular complications in T2DM with NAFLD.

### 3.4 Re‐Clustering Indicates Pericyte Recovery and GDF11 Activation

**FIGURE 4:**
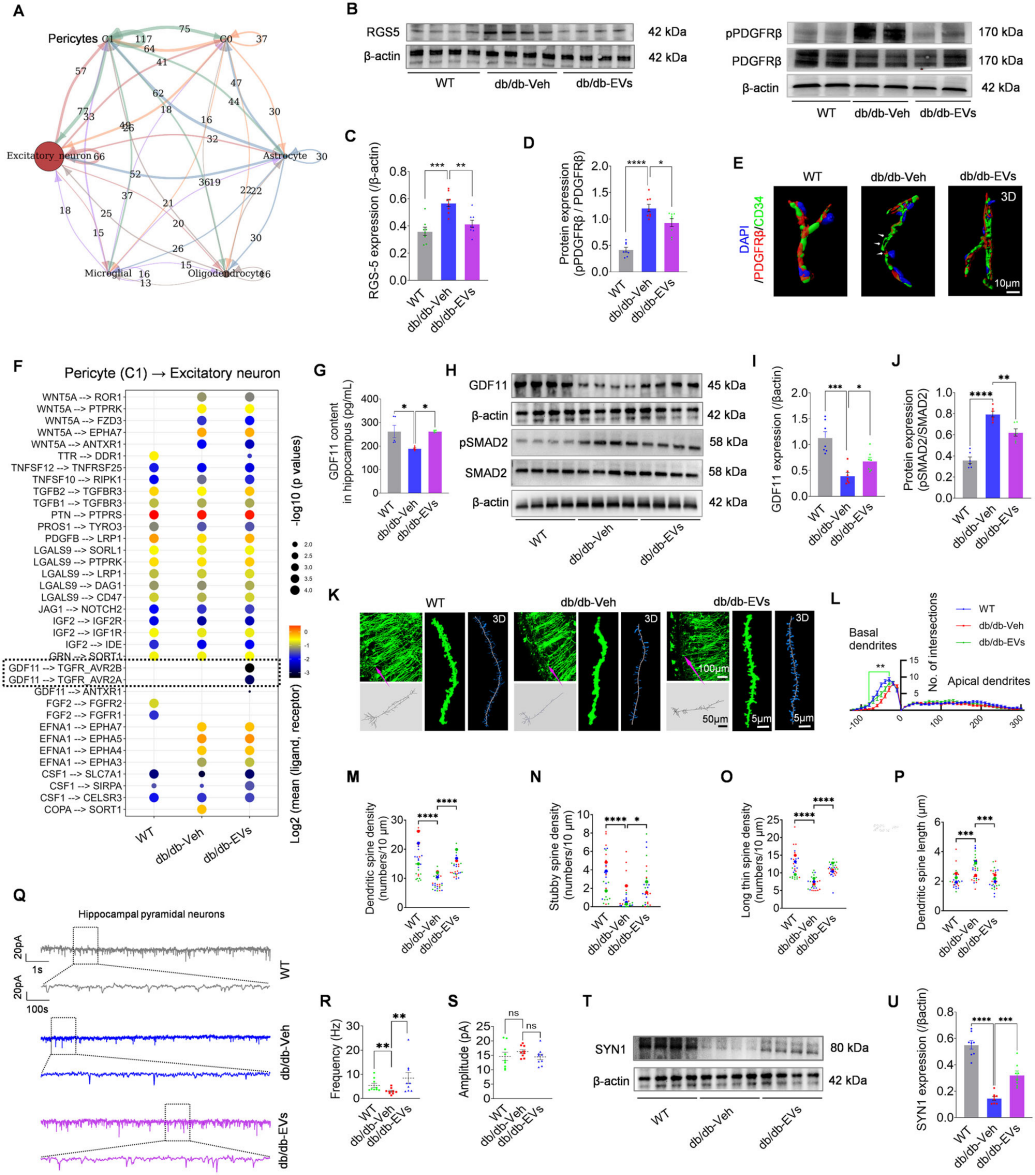
**Pericyte recovery and GDF11 activation in EV‐treated db/db hippocampus**. (A) Network diagram showing the interactions among sub cluster C0, C1, and other cell types. The thickness and colour of the lines represent the strength and type of interactions. CellPhoneDB analysis highlights pericyte (C1)–excitatory neuron interactions in the experimental groups. (B–D) Western blot analysis (B) and quantification of pericyte markers RGS5 (C) and the pPDGFRβ/PDGFRβ ratio (D) demonstrate significant restoration of pericyte function in EVs‐treated db/db mice (*n* = 8). (E) 3D imaging shows PDGFRβ+ pericytes (red) co‐localized with CD34+ endothelial cells (green). White arrows indicate detached pericytes in db/db‐Veh mice, repaired after EV treatment. Scale bar: 10 µm. (F) GDF11‐TGFβ receptor interactions are significantly upregulated in db/db‐EVs‐treated mice, based on statistically enriched ligand‐receptor analysis. (G) ELISA analysis of GDF11 levels in hippocampal tissues of WT, db/db‐Veh, and db/db‐EVs groups (*n* = 5). (H–J) Western blot analysis (H) and quantification of GDF11 (I, *n* = 7) and pSMAD2/SMAD2 (J, *n* = 6) expression in hippocampal tissues of each group. (K, L) Confocal imaging (K) and Sholl analysis (L, *n* = 3 mice, with seven technical replicates) show recovery of dendritic complexity in CA1 pyramidal neurons (excitatory neurons) of db/db‐EVs‐treated mice. Scale bars: 10 µm (overview), 5 µm (magnified). (M–P) Quantification of spine morphology shows increased total spine density (M), stubby spine density (N), long thin spine density (O), and spine length (P) in db/db‐EVs‐treated mice (*n* = 3 mice, with nine technical replicates). (Q–S) mEPSC recordings (Q) and quantification of CA1 pyramidal neurons show significantly increased mEPSC frequency (R), but not amplitude (S), in db/db‐EVs‐treated mice (*n* = 3 mice, with three technical replicates). (T, U) Western blot (T) and quantification of Synapsin I (SYN1, U) show enhanced presynaptic connectivity in db/db‐EVs‐treated mice (*n* = 8). Data are presented as mean ± SEM. “*n*” refers to biological replicates for C, D, G, I, J, U, and to pooled biological and technical replicates for L–P, R, and S. Statistical significance was determined using one‐way ANOVA with Bonferroni post hoc test for (C, D, G, I, J, M–P, R, S, U) and repeated measures ANOVA for (L). Significance levels: ns, not significant (*p* > 0.05); *p < 0.05; **p < 0.01; ***p < 0.001; ****p < 0.0001.

Re‐clustering of Cluster 24 identified three sub‐clusters (C0–C2) defined by highly variable genes and Pseudotime analysis (Figure S6A–C; Table S5). Among these, sub‐cluster C1 emerged as a critical regulatory hub with significant potential to modulate neurophysiology through cellular interactions (Figure 4A). DEG analysis revealed transcriptional changes in sub‐clusters C0 and C1, including remarkable variations in TTR expression (Figure S6D,E). Marker gene analysis confirmed sub‐cluster C1 as pericytes, based on the expression of canonical markers such as regulator of RGS5, PDGFRβ, Kcnj8 and Mcam (Table S6). EV therapy significantly restored pericyte function, as evidenced by restored expression of RGS5 (Özen et al. 2018) and pPDGFRβ (Wang et al. 2023a; Liu et al. 2023) expression in db/db hippocampi (Figure 4B–D). Immunostaining of PDGFRβ and CD34 further demonstrated structural repair of pericytes that had detached from hippocampal vasculature in db/db mice (Figure 4E). These findings indicate that EV therapy promotes pericyte recovery within Cluster 24 of the db/db hippocampus.

To elucidate the mechanism underlying pericyte recovery, ligand‐receptor interaction analysis was performed between pericytes (C1) and excitatory neurons, given their close cellular connectivity (Figure 4A). EV therapy significantly enhanced GDF11‐TGFβ receptor signalling (TGFβR_AVR2A/B), alongside TTR signalling, as revealed by enriched interactions (Figure 4F). This signalling cascade was validated by the activation of SMAD2, a downstream effector of TGFβ signalling (Suragani et al. 2014), in primary hippocampal neurons treated with ACE‐536 (an inhibitor of SMAD2/3) and/or recombinant GDF11 protein (Figure S6F–H). Notably, EV therapy not only elevated GDF11 levels in hippocampal tissues (Figure 4G–I) and pericytes (Figure S6I), but also mitigated pathological SMAD2 activation (Figure 4H,J), which is implicated in diabetic brain dysfunction (Bao et al. 2010). Based on previous studies (Moigneu et al. 2023; Wang et al. 2023b; Cohen et al. 2025), these findings suggest that EV‐induced GDF11 activation contributes to the neurological improvements observed in db/db mice. Supporting these insights, Golgi staining revealed that EV therapy significantly enhanced dendritic complexity and spine density in CA1 pyramidal cells (excitatory neurons) of db/db mice (Figure 4K–P), indicating improved structural plasticity. Electrophysiological recordings further demonstrated increased miniature excitatory postsynaptic current (mEPSC) frequency, reflecting enhanced presynaptic connectivity (Figure 4Q–S). These findings were corroborated by elevated expression of the presynaptic marker SYN1 in hippocampal tissues following EV therapy (Figure 4T,U). Collectively, these results highlight pericyte recovery and GDF11 activation as key contributors to the improvements in neurovascular structure and function observed in the db/db hippocampus following EV therapy.

### 3.5 PDGFB Reduction Links NAFLD Repair to Neurological Improvements

**FIGURE 5:**
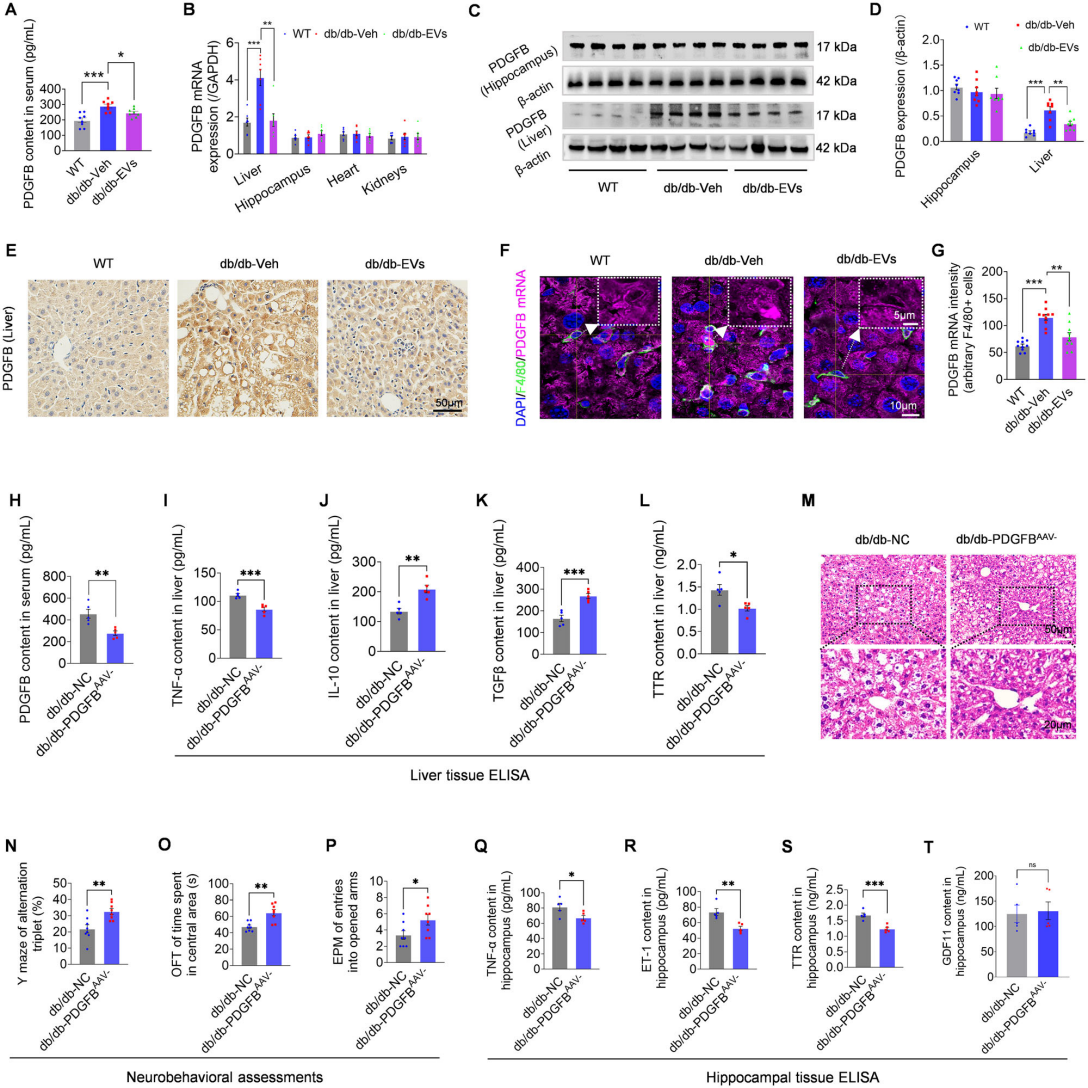
**Effects of PDGFB modulation in the liver and brain of db/db mice**. (A) Serum PDGFB levels measured by ELISA show significant increases in db/db‐EVs mice compared to db/db‐Veh (*n* = 8). (B) qPCR analysis reveals significantly decreased *PDGFB mRNA* in the liver of db/db‐EVs mice, with no changes in other tissues, including the hippocampus, heart and kidneys (*n* = 7). (C, D) Western blot (C) and quantification (D) show selective reduction of PDGFB protein in the liver of db/db‐EVs group, with no changes in the hippocampus (*n* = 8). (E) Representative immunohistochemistry images of liver showing marked changes of PDGFB expression among the experimental groups (*n* = 4). Scale bar: 50 µm. (F, G) Fluorescence in situ hybridization (FISH) images (F) reveal a significant reduction in *PDGFB mRNA* (pink) in liver macrophages (F4/80+, green) of db/db‐EVs mice, as quantified in (G) (*n* = 10 F4/80+ cells from three mice per group). Scale bars: 5 µm (magnified) and 10 µm. (H) ELISA analysis of serum PDGFB levels in db/db mice treated with AAV8‐NC (db/db‐NC) or AAV8‐GP‐1‐PDGFB knockdown (db/db‐PDGFB^AAV−^), confirming successful PDGFB suppression (*n* = 5). (I–L) ELISA measurements of liver TNF‐α (I), IL‐10 (J), TGF‐β (K) and TTR (L) in db/db‐NC and db/db‐PDGFB^AAV−^ groups (*n* = 5). (M) H&E staining of liver section from db/db‐PDGFB^AAV−^ group showing reduced ballooning degeneration and inflammation in the liver, marked portal area, compared to db/db‐NC group. Scale bars: 20 µm (down) and 50 µm (up). (N–P) Neurobehavioral tests reveal improved Y‐maze alternation (N), increased central time in OFT (O), and more open‐arm entries in EPM (P) in db/db‐PDGFB^AAV−^ mice compared to NC mice (*n* = 8). (Q–T) ELISA analysis reveals the changes in hippocampal TNF‐α (Q), ET‐1 (R), TTR (S), and GDF11 (T) levels in db/db‐PDGFB^AAV−^ mice compared to NC groups (*n* = 5). Data are mean ± SEM. “*n*” refers to biological replicates for A, B, D, H‐L, N‐T, and to pooled biological and technical replicates for G. Statistical significance was determined by one‐way ANOVA with Bonferroni post hoc test for (A, B, D, G), unpaired two‐tailed Student's t‐tests (I‐L, Q‐T), and Mann‐Whitney U test for (N–P). Significance levels: ns, not significant (*p* > 0.05); *p < 0.05; **p < 0.01; ***p < 0.001; ****p < 0.0001.

The PDGFB‐PDGFRβ signalling pathway is critical for maintaining pericyte homeostasis (Wang et al. 2023a). To investigate the impact of EV therapy on PDGFB expression, protein and RNA analyses revealed that EV treatment significantly reduced PDGFB levels in the serum and liver (Figure 5A–E), while with no significant changes observed in other organs, such as the hippocampus, heart, and kidneys in db/db mice (Figure 5B–D). FISH analysis confirmed that EV therapy significantly reduced PDGFB released from liver macrophages (Figure 5F,G), key contributors to NAFLD progression (Alabdulaali et al. 2023; Krenkel and Tacke 2017). These findings, combined with the known immunomodulatory effects of EVs (Gou et al. 2022), suggest that EVs regulate macrophage activity to suppress hepatic PDGFB expression. Supporting this, EVs mitigated PA‐induced inflammation in BMDMs and RAW 264.7 macrophages, promoting a shift from the pro‐inflammatory M1 phenotype (CD86, IL‐1β, TNF‐α) to the anti‐inflammatory M2 phenotype (CD206, TGF‐β) (Figure S7A–P). Mechanistically, liver‐tropic AAV8‐mediated PDGFB downregulation in db/db mice (db/db‐PDGFB^AAV−^, Figure 5H) ameliorated hepatic macrophage activity (Figure 5I–K), reduced TTR synthesis (Figure 5L), and improved NAFLD pathologies (Figure 5M). Conversely, PDGFB upregulation in WT (WT‐PDGFB^AAV+^) mice induced NAFLD features, including increased TTR production (Figure S8A–E). Similarly, PDGFB reduction was associated with decreased TTR levels in the liver of MCD mice following EV therapy (Figure S8F–J). Together, these findings suggest that EV‐induced PDGFB reduction is a key contributor to NAFLD repair and TTR regulation.

Beyond hepatic repair, in vitro studies showed that prolonged exposure to high concentrations of PDGFB significantly increased pericyte apoptosis, as indicated by Annexin V/PI staining (Figure S9A,B). This aligns with previous reports that excessive PDGFB disrupts pericyte homeostasis (Liu et al. 2023). The role of PDGFB in pericyte dysfunction was further confirmed by blocking PDGFRβ with AG1296, a PDGFR inhibitor (Contreras et al. 2021), which reduced the activation of PDGFRβ (pPDGFRβ/PDGFRβ ratio) and downstream signalling phosphorylated extracellular signal‐regulated kinase (pERK/ERK ratio) induced by PDGFB supplementation (Figure S9C, D). In vivo, PDGFB^AAV−^ treatment in db/db mice significantly improved neurobehavioral performance (Figure 5N–P) and reduced hippocampal levels of TNF‐α, ET‐1 and TTR (Figure 5Q–S). Similar reductions in hippocampal TTR expression were also observed in EV‐treated MCD mice (Figure S8K). These findings demonstrate that EVs improve neurovascular health by reducing liver PDGFB release and its mediated TTR dynamics in T2DM with NAFLD. Interestingly, modulation of GDF11 expression was not observed in the AAV‐intervened hippocampus (Figure 5T), suggesting that PDGFB suppression alone may not fully account for the broader effects of EV therapy. This highlights the need for future studies to explore additional mechanisms underlying EV‐mediated neurovascular repair.

### 3.6 EV‐miR‐31‐5p Suppresses Hepatic PDGFB Expression

**FIGURE 6:**
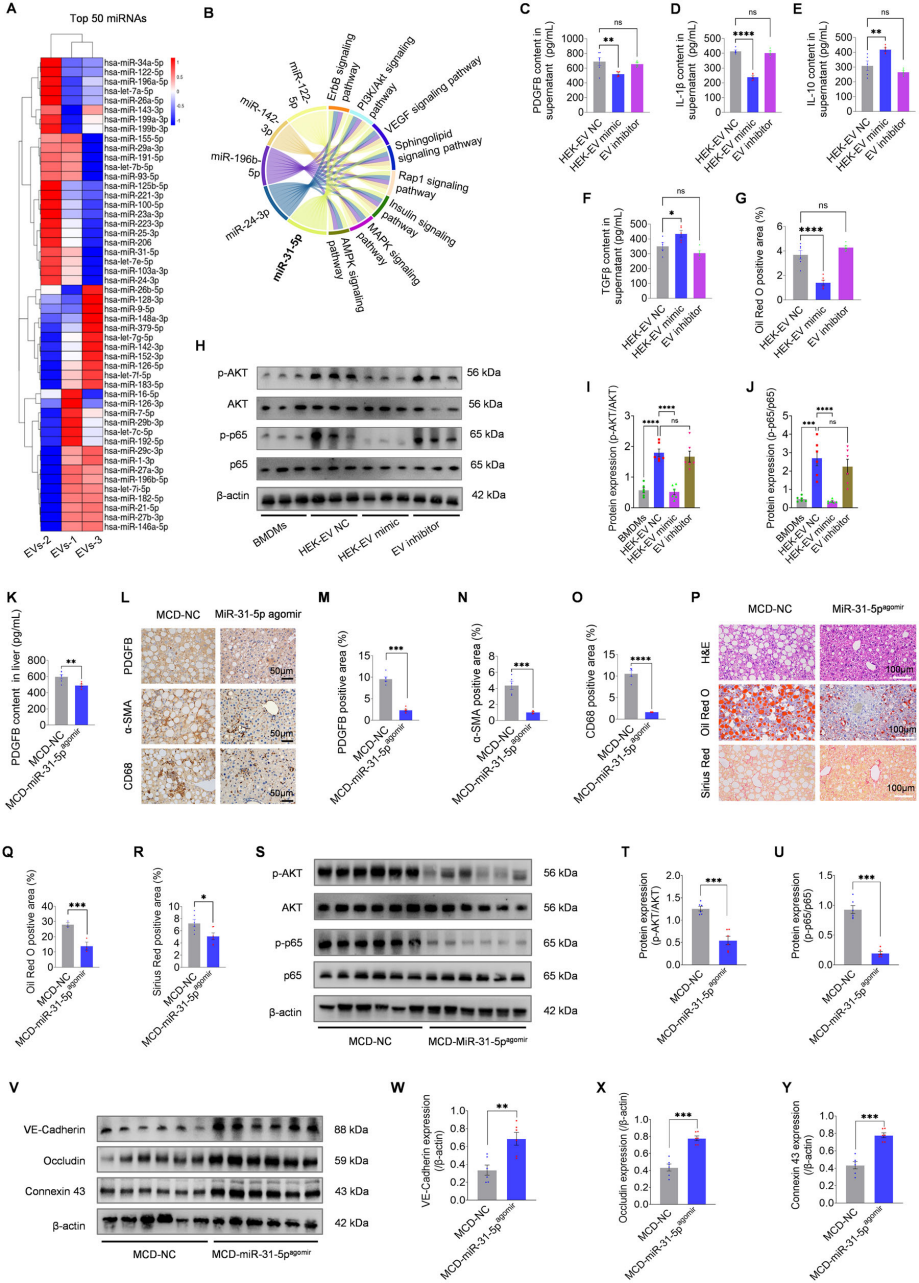
**NGS analysis of EVs and the regulatory role of miR‐31‐5p in severe NAFLD**. (A) Heatmap displaying the top 50 differentially expressed miRNAs identified in EVs from three independent samples (EVs‐1, EVs‐2, EVs‐3). Differential expression analysis using edgeR (FDR < 0.05; |log2(FC)| > 1). (B) KEGG pathway enrichment analysis of these miRNAs revealed significant involvement in immune‐related processes, including mitogen‐activated protein kinase (MAPK) and phosphoinositide 3‐kinase/protein kinase B (PI3K/Akt) signalling. (C–F) ELISA quantification of PDGFB (C), IL‐1β (D), IL‐10 (E) and TGFβ (F) in the supernatant of palmitic acid (PA)‐stimulated bone marrow‐derived macrophages (BMDMs, NASH model) treated with HEK‐EV NC, HEK‐EV mimic, or EV inhibitor (*n* = 5–6). (G) Quantification of Oil Red O‐positive areas (%) in experimental groups (Figure S10G) shows reduced lipid accumulation in the HEK‐EV mimic group, while inhibition of miR‐31‐5p in MSC‐EVs (EV inhibitor) abrogated this effect (*n* = 6). (H–J) Western blot analysis (H) and quantification of PDGFB effectors, including p‐AKT/AKT (I) and p‐p65/p65 (J), in PA‐stimulated BMDMs following treatment with miR‐31‐5p NC, mimic, or inhibitor. Untreated BMDMs served as the negative control (*n* = 6). (K) ELISA quantification of liver PDGFB levels in MCD mice treated with miR‐31‐5p NC or agonist (miR‐31‐5p^agomir^) (*n* = 6). (L‐O) Immunohistochemistry (L) and quantification of PDGFB (M), α‐SMA (N), and CD68 (O) in liver sections from MCD mice treated with NC or miR‐31‐5p^agomir^ (*n* = 5–6). Scale bars: 50 µm. (P–R) Histological analysis of liver sections from MCD mice treated with NC or miR‐31‐5p^agomir^. H&E, Oil Red O, and Sirius Red staining (P) reveal reduced lipid accumulation and fibrosis in the miR‐31‐5p^agomir^ group. Quantification of Oil Red O‐positive (Q) and Sirius Red‐positive (R) areas confirms these improvements (*n* = 6). Scale bars: 100 µm. (S‐U) Western blot analysis (S) and quantification of p‐AKT/AKT (I) and p‐p65/p65 (J) in MCD mice followed by miR‐31‐5p NC or agomir administration (*n* = 6). (V‐Y) Western blot analysis of hippocampal vascular markers (S) and quantification of VE‐Cadherin, Occludin and Connexin 43 in the NC‐ or agomir‐treated mice (n = 6). Data are presented as mean ± SEM. “*n*” indicates the number of experimental replicates (C–G, I, J) or biological replicates (K, M–O, Q, R, T, U, W–Y). Statistical significance was determined using one‐way ANOVA with Bonferroni post hoc tests (C–G) or unpaired two‐tailed Student's *t*‐tests (I–K, M–O, Q, R, T, U, W–Y). Significance levels: ns (*p* > 0.05); *p < 0.05; **p < 0.01; ***p < 0.001; ****p < 0.0001.

EVs are known to carry diverse therapeutic cargoes, including RNAs, proteins and metabolites, which contribute to their multifaceted biological effects (Kalluri and LeBleu, 2020; Cheng and Hill 2022). To elucidate mechanism by which EVs regulate liver‐derived PDGFB, we focused on microRNAs (miRNAs), critical regulators of metabolic and inflammatory pathways (Mori et al. 2019). NGS analysis of EVs revealed 2467 miRNAs, with 173 consistently expressed across three samples (Figure S10A,B; Table S7). Among the top 50 candidates (Figure 6A), miR‐31‐5p and miR‐24‐3p were identified as potential regulators of the PDGF signalling pathway (Table S8). Functional enrichment analysis highlighted their involvement in immune‐related pathways, such as mitogen‐activated protein kinase (MAPK) and phosphoinositide 3‐kinase/protein kinase B (PI3K/AKT) signalling (Figure 6B), which are known to regulate inflammation and fibrosis in NAFLD (Ye et al. 2023; Bai et al. 2024). qPCR confirmed enrichment of these miRNAs in EVs, with miR‐31‐5p emerging as a promising candidate for regulating PDGFB (Table S8).

To investigate the regulatory role of miR‐31‐5p, we employed miRNA‐modified EVs in NASH models, which better reflect the immune responses and fibrosis characteristic of NAFLD (Loomba et al. 2021). *In vitro*, HEK293 (non‐immunomodulatory cells)‐derived EVs loaded with miR‐31‐5p mimic (HEK‐EV mimic; Figure S10D,E) significantly reduced PDGFB expression (Figure 6C; Figure S10F), promoted an M1‐to‐M2 macrophage phenotype shift (Figure 6D–F), and alleviated lipid accumulation in BMDMs under PA stress (Figures S10G and 6G). Conversely, inhibition of miR‐31‐5p in MSC‐EVs (EV inhibitor) neutralized these therapeutic effects compared to HEK‐EV NC groups (Figures 6C–G and S10F, G). Furthermore, we examined downstream effectors of PDGFB, including AKT and NF‐κB (p65), which are implicated in NAFLD progression (Ye et al. 2023; Chu et al. 2025) and highlighted in our KEGG analysis (Figure 6B). HEK‐EV mimic significantly reduced PA‐induced phosphorylation of AKT and p65, whereas the inhibitor had no significant effect (Figure 6H–J). These findings suggest that EV‐delivered miR‐31‐5p suppresses PDGFB signalling and NAFLD‐related responses in cell models. To corroborate these findings, the agonist of miRNA (miR‐31‐5p^agomir^) was used to treat MCD mice, our results showed that miR‐31‐5p^agomir^ administration significantly reduced PDGFB expression (Figure 6K–M) and alleviated NAFLD pathology, as evidenced by reduced α‐SMA and CD68 expression, lipid accumulation, and fibrosis (Figure 6L,N–R). Also, p‐AKT and p‐p65 levels in MCD mouse livers were significantly reduced following miR‐31‐5p agomir treatment (Figure 6S–U). To explore the liver–brain axis, we examined markers of adherens (VE‐Cadherin), tight (Occludin), and gap (Connexin 43) junctions in the hippocampus of recipient mice, and found that miR‐31‐5p agomir restored vascular integrity in MCD mice (Figure 6V–Y). Collectively, these results identify miR‐31‐5p as a key therapeutic modulator within EVs that attenuates PDGFB signalling from hepatic macrophages, thereby alleviating NAFLD pathology and preserving hippocampal health.

## 4 Discussion

This study introduces a novel therapeutic approach utilizing MSC‐EVs to address the interconnected hepatic and neurovascular complications in T2DM with NAFLD. Remarkably, we demonstrate that MSC‐EVs preferentially target the liver, effectively mitigating NAFLD pathology while improving neurovascular integrity in recipient mice. By leveraging the liver‐brain axis (Hadjihambi et al. 2023; Lu et al. 2024b), this intervention establishes a mechanistic framework linking hepatic repair to neurological health through circulated PDGFB modulation, offering an innovative approach to alleviating systemic complications in T2DM with NAFLD.

The liver's central role in systemic homeostasis and cross‐organ communication underpins the mechanism of MSC‐EV therapy (Kim et al. 2024; Lu et al. 2024c). Consistent with prior studies demonstrating organ‐specific biodistribution of EVs (Kang et al. 2021; Chung et al. 2024), we highlight the liver‐tropic nature of MSC‐EVs, where they target macrophages, key drivers of NAFLD pathology (Hammerich and Tacke 2023; Park et al. 2023). By delivering miR‐31‐5p, MSC‐EVs suppress PDGFB expression and promote a phenotypic shift from pro‐inflammatory (M1) to anti‐inflammatory (M2) macrophages. This contributes to reduced liver inflammation, fibrosis and steatosis, as well as restoration of metabolic homeostasis. Crucially, modulation of the PDGFB‐PDGFRβ axis extends therapeutic benefits to the brain (Liu et al. 2023; Okekawa et al. 2024). Downregulating PDGFB enhances hippocampal pericyte recovery and neurovascular integrity, stabilizing the blood‐brain barrier (BBB) and improving cognitive outcomes (Sweeney et al. 2016; Nation et al. 2019). These findings align with growing evidence linking hepatic dysfunction to neurovascular complications *via* systemic circulating factors (Okekawa et al. 2024), and underscore the interconnected nature of metabolic and neurovascular pathology in T2DM with NAFLD.

Moreover, EV‐mediated suppression of PDGFB also impacts transthyretin (TTR) dynamics, which primarily synthesized in liver and play a dual role in neuroprotection and reducing pathological deposition in the diabetic hippocampus (Magalhães et al. 2021; Adams et al. 2023). Specifically, our results reveal upregulation of TTR mRNA in hippocampal cells after EV treatment, suggesting a compensatory neuroprotective mechanism that restores homeostasis (Magalhães et al. 2021). Interestingly, reduced protein levels of TTR in serum and hippocampus may reflect adaptive post‐transcriptional regulation or accelerated protein turnover, thereby minimizing pathological accumulation while retaining its protective functions (Ruberg and Maurer, 2024). Additional pathways, such as GDF11 activation, further contribute to enhanced neuroplasticity and vascular stability (Moigneu et al. 2023; Wang et al. 2023b; Schafer and LeBrasseur, 2019). Collectively, these mechanistic insights reinforce the potential of MSC‐EVs to act as a bridge between liver repair and systemic neurovascular improvements, illustrating their transformative capabilities as a cross‐organ therapeutic tool and filling a critical gap in the field where multi‐organ interactions are often overlooked.

Our findings provide valuable insight for the advancement of EV‐based therapies. While EV‐miR‐31‐5p regulates hepatic PDGFB signalling and shows therapeutic potential, advanced reporter assays are needed to further elucidate its direct targets. Additionally, the observed systemic outcomes, such as GDF11 activation and cerebrovascular recovery, point to the likely involvement of other EV cargo. Advanced molecular profiling, including single‐vesicle RNA sequencing, will be essential for further unravelling the complexity of EV‐mediated effects (Carney et al. 2025; Kaur et al. 2022). Regarding EV isolation, ultracentrifugation (UC) remains a widely used and effective method (Thery et al. 2006; Thery et al. 2018), and yielded functionally active EVs in our study. However, UC may co‐isolate contaminants (Correll et al. 2022), while size‐exclusion chromatography (SEC) and density‐gradient UC can improve purity, their application can be limited by equipment availability or lower yield (Carney et al. 2025; Welsh et al. 2024). Therefore, further optimization of isolation methods to balance purity and yield will be key for clinical translation of EV‐based therapies. Finally, future research should extend MSC‐EV investigations to address other T2DM‐related organ complications, such as those affecting the heart and kidneys, in order to facilitate the development of effective multi‐organ therapies.

In conclusion, our findings establish EVs as a transformative cross‐organ therapeutic platform that leverages the liver‐brain axis to address the intertwined metabolic and neurovascular complications of T2DM with NAFLD. By targeting hepatic macrophages and modulating PDGFB and TTR dynamics, EVs provide a precision‐targeted approach for comprehensive systemic repair. These insights lay the groundwork for the development of next‐generation EV‐based nanotherapeutics designed to combat systemic dysfunction in metabolic disorders.

## Author Contributions

**Minghao Du**: conceptualization, methodology, software, data curation, formal analysis, investigation, writing–original draft. **Hao Yang**: methodology, formal analysis, data curation, investigation, writing–original draft. **Jinyun Niu**: methodology, investigation, visualization, formal analysis, writing–original draft. **Jing Huang**: methodology, investigation, visualization, formal analysis, writing–original draft. **Lihong Wang**: conceptualization, methodology, visualization, supervision. **Junxiu Xi**: methodology, visualization, data curation, formal analysis. **Panpan Meng**: methodology, visualization, formal analysis. **Zhiyong Liu**: methodology, visualization, formal analysis. **Guaiguai Ma**: methodology, formal analysis, data curation. **Jiani Li**: methodology, data curation. **Xiaoyan Liu**: methodology, visualization, formal analysis. **Liang Guo**: visualization, data curation, formal analysis. **Mingjun Hu**: visualization, data curation, formal analysis. **Zhufang Tian**: validation, data curation, formal analysis. **Bin Liu**: methodology, data curation. **Weiping Liu**: conceptualization, supervision, project administration. **Ashok K. Shetty**: supervision, writing–review and editing. **Shengxi Wu**: project administration, supervision, writing–review and editing. **Andrius Baskys**: supervision, writing–review and editing. **Qianfa Long**: conceptualization, investigation, funding acquisition, project administration, supervision, writing–original draft, writing–review and editing.

## Conflicts of Interest

The authors have declared that no competing interest exists.

## Supporting information




**Supporting Table S1**: Cell information from snRNA‐Seq data.

**SupportingTable S2**: Marker genes for clusters 0–37.

**SupportingTable S3**: Composition analysis of cell types and clusters.

**SupportingTable S4**: Differentially expressed genes (DEGs) in cluster 24, astrocytes, and excitatory neurons.

**Supporting Table S5**: Sub‐cluster cell information.

**Supporting Table S6**: Marker genes for sub‐clusters.

**Supporting Table S7**: EV‐associated miRNAs compared with Vesiclepedia data.

**Supporting Table S8**: EV‐miRNAs targeting PDGF signaling pathways.

Detailed methods.
Figure S1–S10.

## Data Availability

The datasets from this study have been deposited in the NIH National Library of Medicine and can be accessed using the following accession codes: PRJNA1180752: snRNA‐Seq (hippocampus); PRJNA1181240: mNGS (EVs); PRJNA1181238: EV‐miRNA analysis. Any additional information required to reanalyse the data reported in this paper is available from the lead contact upon request.
