# Supplementary material for: Umbilical Cord‐Mesenchymal Stromal Cell‐Derived Extracellular Vesicles Target the Liver to Improve Neurovascular Health in Type 2 Diabetes With Non‐Alcoholic Fatty Liver Disease
Source: J Extracell Vesicles. 2025 Jul 7;14(7):e70125. doi: 10.1002/jev2.70125 (PMC12230362; doi:10.1002/jev2.70125)
Supplement: Supplementary file 9 — Detailed methods. Figure S1–S10. [file JEV2-14-e70125-s004.docx]

**Supporting information (SI)**

**Detailed Methods**

**Ethics statement**

Human umbilical cord tissues were donated by healthy full-term mothers (ID: 817055; 757878; 819036; 825467; 825512). Their guardians provided written informed consent for the use of samples. All procedures were approved by the Ethics Committee of Xi’an Central Hospital, Xi’an Jiaotong University (Approval No. LAS-L-2022-004-01, Date of approval: Mar 21, 2022), and complied with the standards of the National Health Research Institute. We are committed to adhering to the principles of the 3Rs (Replacement, Reduction, and Refinement) in all our animal research, the experimental procedures were performed in accordance with the Guide for the Care and Use of Laboratory Animals (8th edition, 2011) and approved by the ethical committees of Xi’an Jiaotong University Health Science Center (Approval No. 2021-1104, Date of approval: Feb 20, 2021). All cell samples, tissue materials, adeno-associated viral vectors, recombinant proteins and other biological agents were handled at Biosafety level 2 and approved by Biosafety Committee of Xi’an Central Hospital (Approval No. BCA-2021-0212-001, Date of approval: Feb 12, 2021).

**Animals**

C57BL/6 mice, including pregnant females (embryo at 13-14 days) and adult males (6-8-week-old), were purchased from the Experimental Animal Center of Xi’an Jiaotong University. 16-week-old of male leptin receptor mutant BKS db/db (db/db) mice, male wild-type littermate control BKS db (lean +/+) (WT), 8-week-old of spontaneously hypertensive rats (SHR) and Wistar rats were purchased from Charles River Laboratories (MA, USA). They were housed in groups (3-5 animals per cage) and were allowed a period (2-4 weeks) to acclimatize to the laboratory environment before use. C57BL/6 mice were fed with methionine and choline deficient diet (MCD; ReadyDietch, China) and high fat diet (HFD; ReadyDietch, China) with 60 mg/kg streptozocin (STZ; Solarbio, China) to induce NASH [Zhang, et al. 2019] and T2DM [Kojima, et al. 2003] models, respectively. High-fat diet/streptozotocin (HFD-STZ) mice with glucose levels of ≥16.7mmol/L were selected for the experiments. All experimental animals were housed under specific pathogen-free (SPF) conditions and maintained at a controlled temperature of 22°C to 24°C, with a 12-hour light-dark cycle. They were provided with food and water ad libitum. Additionally, the db/db mice received a controlled diet (4~5g, per day per mouse) after 20 weeks of age to prevent death caused by hyperglycemia and obesity.

**Primary culture of cells**

Primary cultures of MSCs were isolated from Wharton’s jelly in the human umbilical cord and cultured in 175 cm² flasks with complete culture medium (CCM), consisting of α-minimum essential medium (α-MEM; Thermo Fisher Scientific, USA) supplemented with 16.5% fetal bovine serum (FBS; Sigma-Aldrich, USA). Cells were seeded at a density of 2–5 × 10⁴ cells/cm² and maintained at 37°C in a 5% CO₂ incubator, as previously described [Xian, et al. 2019; Wang, et al. 2020]. Cell characterization was performed using flow cytometry for surface antigens including CD105, CD90, CD73, CD45, and CD34, these antibodies were purchased from BioLegend (CA, USA) (Data were not shown).

Considering their essential role in NAFLD progression [Kazankov, et al. 2019], bone marrow-derived macrophages (BMDMs) were collected from the bone marrow of femurs and tibias and cultured with RPMI-1640 (Thermo Fisher Scientific, USA) containing 10% FBS and 20 ng/mL recombinant murine macrophage colony-stimulating factor (M-CSF, PeproTech, USA) for 7 days according to previous protocol [Ying, et al. 2013]. Bone marrow tissues were harvested from 8-week-old male C57BL/6J mice, and the purity of these cells was identified by flow cytometry analysis of F4/80 (BioLegend, USA) and CD11b (BioLegend, USA). BMDMs were used because they are an established in vitro model that reflect the behavior of liver-resident and recruited macrophages in vivo, which are central to NAFLD pathogenesis [Kazankov, et al. 2019]. To induce NASH cell model, BMDMs were stimulated with 0.6 mmol/L palmitic acid (PA, Kunchuang Biotechnology, China) for 24 hours as previously described [Ni, et al. 2022].

Hippocampal neurons were isolated from mouse embryos (embryonic day 17~18), as described previously [Luo, et al. 2021]. The harvested cells were cultured with neurobasal medium (Thermo Fisher Scientific, USA) supplemented with 2% B27 (Thermo Fisher Scientific, USA), 100 U/mL penicillin-streptomycin (Novnature, China), and 2 mM L-glutamine (Thermo Fisher Scientific, USA). The purity of hippocampal neurons was assessed by immunofluorescence staining using NeuN (Abcam, USA) and MAP2 (Abcam, USA) as a neuronal marker. For the growth differentiation factor 11 (GDF11) intervention, 40 ng/mL of recombinant GDF11 protein (PeproTech, USA) was added to the medium, with or without 1 µg/mL Luspatercept (ACE-536) (a Smad2/3 inhibitor; MCE, USA) for 2 hours, as described in previous reports [Moigneu, et al. 2023; Suragani, et al. 2014]. All primary cultures were evaluated for viability by trypan blue exclusion and counted using an automated cell counter (JSY-SC-031, BodBoge, China). Only those with >95% viability were used in experiments.

**Cell lines**

Human bone marrow-derived macrophages (hBMDMs), human brain microvascular pericytes, human embryonic kidney 293 (HEK293) cells, and RAW 264.7 cells (mouse monocyte macrophages) were obtained from ScienCell Research Laboratories (CA, USA). These cells were cultured in Dulbecco's Modified Eagle Medium (DMEM; HyClone, USA) supplemented with 10% FBS and maintained in a humidified atmosphere at 37°C with 5% CO₂. An immune injury cell model was induced by treating hBMDMs with 100 ng/mL lipopolysaccharide (LPS; Sigma-Aldrich, USA) for 24 hours [Zhao, et al. 2024]. To investigate whether prolonged elevated levels of platelet-derived growth factor subunit B (PDGFB) in serum cause pericyte damage, varying concentrations of PDGFB recombinant protein (MCE, USA) (10, 50, 100 ng/mL) were added to the culture medium as previous reports [Gu, et al. 2021]. The cells were incubated for different durations (4, 8, 24, and 48 hours), with ddH2O serving as the solvent control. At each time point, flow cytometry was used to assess the apoptosis ratio. To elucidate PDGFB-PDGFRβ signaling in pericytes, the agonist group was treated with PDGFB at a concentration of 20 ng/mL. In the inhibitor group, pericytes were first pre-treated with AG1296 (a PDGFR tyrosine kinase inhibitor; CA, USA) at 10 μM for 1 hour, after which PDGFB was added at 20 ng/mL, and the cells were cultured for 24 hours. AG1296 at 10 μM was also utilized as a negative control group, as previously reported [Gu, et al. 2021]. Additionally, RAW 264.7 cells were treated with 0.6 mmol/L palmitic acid (PA) for 24 hours to establish NASH cell model, as previously described [Ni, et al. 2022].

**EV isolation**

The third to fifth passages of MSCs were cultured at a concentration of 2–4 × 10⁵ cells/ml in α-MEM supplemented with 10% EV-depleted FBS for 48 hours [Kornilov, et al. 2018]. Each 175 cm² flask contained 25 ml of medium, corresponding to approximately 1.5–2.5 × 10⁷ cells per flask. A total of 2000 mL of conditioned medium was collected for further analysis. Afterward, cell supernatants were collected and subjected to sequential centrifugation using a ST16R centrifuge (Thermo Fisher Scientific, USA), as previously described [Zhao, et al. 2022; Thery, et al. 2006]. After which, syringe-filter the supernatant through 0.22 µm filter. These supernatants were then further centrifuged at 100,000 × g for 90 minutes with an XPN-100 ultracentrifuge (Beckman Coulter, USA) to obtain the EV pellets. The collected pellets were resuspended in sterile PBS (Gibco, USA) and subjected to an additional centrifugation at 120,000 × g for 70 minutes to eliminate free proteins and impurities. Also, crude EVs were isolated by centrifuging the cell supernatant at 100,000 × g for 1.5 hours. The pellet was resuspended and loaded onto a sucrose gradient (10–82%, 1.002–1.34 g/ml density) for centrifugation at 100,000 × g for 16 hours. Six density fractions were collected, diluted in PBS, and centrifuged again at 100,000 × g for 1.5 hours to pellet purified EVs [Crewe, et al. 2018; Thery, et al. 2018]. The protein content of the EV suspension was quantified using a bicinchoninic acid (BCA; Beyotime, China) assay, following the manufacturer's instructions. These isolated EVs were stored at -80°C until further use.

**Quality control of EVs**

Quality control of EVs derived from MSCs (donor samples 1~5) was performed using metagenomic next-generation sequencing (mNGS) by Hugobiotech Co., Ltd. (Beijing, China), as previously documented [Lu, et al. 2024]. Briefly, DNA was extracted and purified from 200 μL (approximately 150 μg) of MSC-EVs according to the manufacturer's instructions for the QIAamp DNA Micro Kit (Qiagen, Germany). The DNA concentration and quality were assessed using Qubit Fluorometric Quantitation and agarose gel electrophoresis. DNA libraries were prepared using the QIAseq™ Ultralow Input Library Kit (Qiagen, Germany). The library concentration and quality were confirmed through Qubit and agarose gel electrophoresis. Libraries, each tagged with a unique barcode, were pooled and sequenced on an Illumina NextSeq platform (Illumina HiSeq X10, Illumina, USA). Sequencing data underwent quality control to eliminate adapter sequences, low-quality reads, low-complexity reads, and short reads. Human reads were filtered out by mapping against the human reference genome using the SNAP software tool. The filtered reads were then aligned to a microbial genome database using the Burrows-Wheeler Alignment (BWA) tool. This database included over 20,000 microbial genomes sourced from NCBI, comprising 11,910 bacterial, 7,103 viral, 1,046 fungal, and 305 parasitic genomes. Finally, the microbial compositions of the samples were determined and analyzed using mNGS. The microbial data were deposited in the NIH National Library of Medicine (accession number: PRJNA1181240).

**EV characterization and activity test**

Three EV preparations passed quality control (Figure S1A), and purified EVs (P-EVs) were characterized using Western blotting to detect representative markers. These markers included CD81, CD63, CD9, and TSG101 as positive indicators, and Calnexin as a negative control to confirm the absence of cellular contaminants [Witwer, et al. 2019]. The detailed information of these antibodies was provided in the Table 1. The morphology and size distribution of these EVs were analyzed using transmission electron microscopy (TEM; JEM-1400, JEOL Ltd., Japan) and nanoparticle tracking analysis (NTA; ZetaVIEW S/N 17-310, Particle Metrix, Germany). To evaluate the immunomodulatory activity of these EVs, hBMDMs were pretreated with 10 μg/mL EVs and P-EVs for 12 hours to ensure uptake [Zhao, et al. 2022], followed by stimulation with 100 ng/mL LPS for 24 hours (hBMDMs-EVs-LPS; hBMDMs-P-EVs-LPS) according to recent report [Zhao, et al. 2024]. Untreated hBMDMs served as the negative control, while LPS-stimulated hBMDMs with PBS addition served as the model control (hBMDMs-LPS). The cell supernatants were collected and analyzed using ELISA kits for TNF-α, iNOS, IL-4, and IL-10 (Table 1), as per the manufacturer’s instructions.

**EV Labeling**

To track EVs *in vivo*, the EVs were first incubated with 0.01% tin(II) chloride dihydrate (Sigma, USA) on a shaker for 5 minutes. Subsequently, various doses of technetium-99m (_99m_TcO4_⁻_; Guangdong CI Medicine Co., Ltd., China) (111, 139.5, 148, 166.5, and 185 MBq) were added. The mixtures were incubated at room temperature for 30 min and then purified using PD MiniTrap Sephadex G-25 columns (Cytiva, Wash. D.C., USA), as previously described [Chung, et al. 2024; Yang, et al. 2024]. An aliquot was applied to ITLC-SG (Gelman Sciences, USA) chromatography paper and eluted with 100% acetone. Labeling efficiency was quantified by a radioactive thin-layer imaging scanner (Scan-RAM MCA, USA) to confirm the criteria were met. Cell viability was assessed using the Cell Counting Kit-8 (CCK8; New Cl & Molecular Biotech Co. Ltd., China), which showed no significant impact of ^99m^Tc labeling on the ability of EVs to regulate cell growth and proliferation in BMDMs and RAW 264.7 cells. The stability of the ^99m^Tc-labeled EVs was evaluated by measuring the percent change in radiochemical purity over time (0–24 hours). Based on labeling and stability results, 148 MBq of ^99m^TcO4_⁻_ was used to label 100 µg of EVs. For *in vivo* use, the final mixture contained 50 µg EVs in 150 µL, with a technetium-99m dose of 74 MBq. For ex vivo tracking, the vesicles were labeled with the red fluorescent membrane dye PKH26 (Sigma-Aldrich, USA) and EV marker CD9 (Abcam, USA), following established protocols [Long, et al. 2017; Lu, et al. 2025].

**EV-miRNA analysis via NGS**

Total RNA was extracted from plasma using the Qiagen Serum/Plasma Kit (Qiagen, Hilden, Germany). RNA quality and integrity were assessed using the Agilent 2100 BioAnalyzer with the High Sensitivity DNA Kit (Agilent Technologies, Santa Clara, CA, USA). NGS libraries were prepared with the QIAseq miRNA Library Kit (Qiagen, Hilden, Germany), involving sequential adapter ligation to the 3′ and 5′ ends of miRNAs, UMI-based cDNA synthesis, cDNA cleanup, and amplification with a universal forward primer and indexing reverse primers. Library quality was evaluated with the Agilent 2100 BioAnalyzer system. Sequencing was conducted on an Illumina NextSeq 6000 platform (Illumina, San Diego, CA, USA) at Shanghai Biotechnology Corporation (Shanghai, China). After sequencing, low-quality and contaminant reads were removed using the Fastx Toolkit (v0.0.13). Clean reads were mapped to the GRCh38 human reference genome with Bowtie, and known miRNAs were identified via miRBase (release 21), while novel miRNAs were predicted using miRCat. Gene expression levels were quantified as transcripts per million (TPM). Differentially expressed miRNAs were identified using edgeR with an FDR <0.05 and |log2 fold change| >1. Target genes were predicted using miRanda, and functional characterization was performed via Kyoto Encyclopedia of Genes and Genomes (KEGG) pathway enrichment analyses. The sequencing data were submitted to the NIH National Library of Medicine database (accession number: PRJNA1181238).

**EV administration**

EV batches were randomly selected, ensuring that each biological replicate included EVs derived from all three preparations to account for potential variability among batches. MSC-EVs were administered at a dose of 2 mg of protein per kilogram of body weight (equating to approximately 10^12^ vesicles per kilogram). The EVs were diluted in 150 μL of sterile PBS, following previous recommendations [Xian, et al. 2019; Cheng, et al. 2023]. Considering the high metabolic rate and obesity in db/db mice, EV treatment was delivered through repeated injections to maximize absorption and reduce mortality. The total dose of 2 mg of protein/kg was administered in divided doses at 20 and 21 weeks of age (db/db-EVs). Methionine and choline-deficient (MCD) diet mice were administered a single injection of EVs at a dosage of 2 mg of protein per kilogram of body weight (MCD-EVs). The intervention time points for db/db (20 weeks old) and MCD (12 weeks old) mice were primarily chosen to enhance the reversal effect of EVs on MAFLD, as supported by existing literature [Yang, et al. 2020; Zou, et al. 2024]. An equivalent volume of PBS was administered via tail vein injection as a vehicle control (db/db-Veh or MCD-Veh).

To track the MSC-EVs in vivo, 20-week-old db/db mice, 12-week-old MCD mice, 20-week-old HFD/STZ mice, and age-matched WT or C57BL/6 mice received intravenous injections of 50 μg of ^99m^Tc-labeled EVs diluted in 150 μL of PBS via the tail vein. SHR and Wistar rats, at 10 weeks of age, were administered 100 μg of EVs dissolved in 300 μL of PBS intravenously. To study EV aggregation via different administration routes, 20-week-old WT mice received an intranasal dose of 30 μg of ^99m^Tc-labeled EVs in 30 μL of PBS, based on a previous study [Long, et al. 2017]. An equivalent dose of free technetium (^99m^TcO4-) was injected as a control in the tracking experiments.

**SPECT/CT imaging**

SPECT/CT (870DR, General Electric Company, USA) imaging was performed to evaluate the biodistribution of EVs in vivo, as previously described [Yang, et al. 2024]. The rodents were anesthetized with isoflurane (3% for induction and 1% for maintenance) to ensure stability. Each group underwent a 1200-second SPECT planar scan. The SPECT/CT imaging used a 256×256 acquisition matrix with a 1.5 zoom factor, capturing each tomographic angle for 20 seconds and CT scans with a 1 mm sectional thickness. Gamma camera (Mediso nanoScan SPECT/CT scanner, Hungary) images were taken at 1-, 6-, 24-, 48-, 72-, and 96 hours post-injection.

**Pharmacokinetics analysis of infused EVs**

A comprehensive pharmacokinetic analysis was performed to evaluate the temporal distribution of 99mTc-labeled EVs in vivo. The study began with radiation measurements using a water model that mimicked the volume and total dose administration in rodents [Uhl, et al. 2015]. Subsequent evaluations in live rodents incorporated both physical (1/T) and biological half-lives (1/Tb), utilizing the effective half-life equation (1/Te = 1/T + 1/Tb) to ensure precise calculation of biological half-life, residual drug quantities, and excretion rates at various intervals. The systemic drug residue percentage was calculated using the formula: Systemic drug residue (%) = [e^⋀^(-ln2t/Te)]×100.

SPECT/CT fusion imaging was employed to map radiation distribution across major organs, such as the brain, liver, lungs, heart, kidneys, spleen, and bladder. A slice thickness of 1 mm in the coronal plane was used to capture maximum organ slices on CT images, allowing for precise boundary delineation and accurate SPECT radiation measurement. This facilitated estimation of organ-specific drug concentrations and their respective percentages of the total body drug load. For muscle and bone assessment, the artificial intelligence (AI) analysis (Q.Volumetrix MI, General Electric Co., USA) was carried out to map out the extent of bone and muscle throughout the body and calculate the radiation dosage. Tissue dissections at selected time points provided isolated organ samples for planar SPECT scanning, validating in vivo measurements. The ex vivo results showed no significant deviation from in vivo calculations, confirming the accuracy and feasibility of the method. Throughout the study, the quantity of EVs in the soft tissue of mice remained at the minimum detection level, with no significant variation in EV quantity per unit weight across different time periods, recorded at 0.163±0.007/g. Therefore, this value was regarded as the background level for soft tissue. The whole-body background level was calculated to be 4.3±0.02 μg.

**AAV production and injection**

Recombinant liver-targeted AAV vectors, namely AAV8-GP-1-PDGFB, AAV8-GP-1-SJ-negative control (NC), AAV8-GP-PDGFB, and AAV8-GP-13N-NC, were synthesized by GenePharma (Shanghai, China) (Table 1). These vectors, dissolved in sterile PBS, were quantified by qPCR to ensure accurate viral genome counts for dosing. The db/db mice received tail vein injections of either AAV8-GP-1-PDGFB (db/db-PDGFB^AAV-^) or AAV8-GP-1-SJ-NC (db/db-NC) to downregulate PDGFB in the liver, with 1×10¹² viral genomes per mouse administered in 50 μL PBS at both 18 and 19 weeks of age. WT mice were injected via the tail vein with either AAV8-GP-PDGFB (WT-PDGFB^AAV+^) or AAV8-GP-13N-NC (WT-NC) to upregulate PDGFB in the liver, using the same dose at 19 weeks of age, following the manufacturer's protocol. Strict sterile techniques and controlled environmental conditions were maintained to prevent contamination and ensure experimental integrity. Data collection occurred when the mice were 24 weeks old, ensuring consistency and reliability throughout the study.

**Pathological staining**

Following the initial administration of EVs or PBS, db/db (2-, 4-, 6-, 8-weeks), MCD (2-, and 4-weeks), and age-matched WT or Naïve (C57BL/6) mice were anesthetized with 3% isoflurane/air mixture inhalation. Liver tissues from sacrificed mice were excised to a size of about 2 cm × 2 cm × 1 cm. For hematoxylin-eosin (H&E) or Sirius red staining, tissues were fixed in 4% paraformaldehyde and embedded in paraffin. Sections (5 μm thick) were prepared and stained with H&E (MCE, USA) or Sirius Red (MCE, USA). For Oil Red O staining, liver tissues were embedded in biological glue, and 10-μm-thick frozen sections were treated with 70% ethanol and stained with Oil Red O (Abcam, USA) according to the manufacturer’s instructions. Additionally, NASH cell models, either treated with EVs or left untreated, were subjected to Oil Red O staining to assess lipid accumulation. Images were captured using a microscope (Leica DMi8, Germany). For statistical accuracy, three random fields were selected and imaged at 200× magnification from each sample in every group. Image analysis was performed using ImageJ Pro Plus V6.0 (Bethesda, Maryland, USA).

**Neurobehavioral tests**

Neurobehavioral tests, including the Y-maze, Open Field Test (OFT), and Elevated Plus Maze (EPM), were performed on db/db mice at 2-, 4-, and 6- weeks post-infusion, MCD mice at 2- and 4- weeks post-treatment, either db/db-PDGFBAAV- or db/db-NC groups, and age-matched vehicle control, as previously described [Liu, et al. 2021; Zhang, et al. 2021]. For the Y-Maze test, the Y-maze, consisting of three equal arms (33 cm long, 10 cm wide, 11 cm high) at 120° angles (labeled 1, 2, and 3), was used to assess spatial memory and locomotor activity. Mice were placed at the distal end of arm 1 and allowed to explore for 8 minutes. The maze floor was cleaned with 10% ethanol between trials. For the OFT, mice were placed individually in a square box (40 cm × 40 cm × 40 cm) with defined central (20 cm × 20 cm) and peripheral areas. Each trial lasted 10 minutes. For the EPM, the apparatus, elevated 50 cm above the ground, consisted of two open arms (30 cm long and 5 cm wide) and two closed arms (30 cm long, 5 cm wide, 11 cm high) arranged perpendicularly from a central platform. Mice were placed on the central platform facing an open arm to begin the test. Behavior was analyzed using SMART v3.0 software (Panlab, USA), measuring parameters such as alternation triplet, speed, time spent in central area, and entries in to open arms. Alternation triplet in Y maze was calculated as: % Alternation = (Number of Alternations / (Total number of Arm Entries - 2)) × 100.

**Transmission electron microscope**

After receiving EVs or PBS for four weeks, the db/db and WT mice were deeply anesthetized and perfused with chilled PBS, followed by a 3% glutaraldehyde solution (Sigma-Aldrich, USA). The brain hippocampi were isolated and processed as previously described [Yang, et al. 2024]. Hippocampal sections were stained with methylene blue (Sigma-Aldrich, USA), while ultrathin sections (60-90 nm thickness) were cut with a diamond knife and stained with uranyl acetate (Sigma-Aldrich, USA) and lead citrate (Sigma-Aldrich, USA). Sections were examined using a transmission electron microscope (HT7700-SS, Hitachi TEM system, Japan).

**SnRNA-Seq analysis**

Hippocampi were collected from db/db mice 4 weeks post-injection with either EV or PBS, with WT mice as controls (n = 3, the hippocampi from two mice in each group were pooled into a single sample). All procedures were conducted on ice within a 4°C ultra-clean workbench. Nuclei were isolated from fresh-frozen tissue using a modified protocol [Nelson, et al. 2023]. Briefly, hippocampal tissue was homogenized in chilled Nuclei EZ Lysis Buffer (Sigma-Aldrich, USA) using a glass dounce tissue grinder (FIS#K885300-0002, Kimble Kontes, USA). Homogenates were filtered through 70 μm strainers, centrifuged at 500 g for 5 minutes at 4°C, or alternatively, cleaned via density gradient centrifugation employing debris removal solution (Miltenyi Biotec, Germany). Resuspended nuclei underwent further centrifugation and filtration through a 35 µm strainer. Approximately 6000 nuclei per sample were captured using 10× Genomics Single Cell 3′ Reagents kit. Sequencing was performed on an Illumina NovaSeq 6000 with 150 bp paired-end reads by Genechem (Shanghai, China). The Cell Ranger Analysis Pipeline (v6.0.2) facilitated the generation of sequencing libraries, and resulting unique molecular identifier (UMI) count matrices were processed using Scanpy (v1.8). Quality control eliminated cells with high mitochondrial content (PMC ≤ 5%), low UMI counts (≤ 100,000), and outside the 200-8,000 gene range. A total of 53,307 single-cell transcriptomes were included for analysis.

Library size normalization was achieved using Scanpy's pp.normalize_ total, followed by a logarithmic transformation (pp.log1p) [Wolf, et al. 2018]Variable genes were identified with Scanpy's pp.highly_ variable_genes [Wolf, et al. 2019], and dimensionality reduction via Principal Component Analysis (PCA) was executed using Scanpy's tl.pca function. Cellular clustering, based on gene expression profiles, was enabled using the pp.neighbors function, with clusters visualized in both 2D and 3D using Uniform Manifold Approximation and Projection (UMAP; tl.umap). Comparative clustering analyses assessed proportional shifts in cell populations across treatment conditions, with re-clustering of Cluster 24 enhancing resolution. Pseudotime analysis elucidated lineage progression, particularly among sub-clusters 0-2. Marker gene identification employed Seurat's FindAllMarkers function, using the Wilcoxon test with adjusted p-value < 0.01 and |fold change| > 2 thresholds. Functional enrichment analyses correlated markers with pathways from GO, HALLMARK, and Reactome databases via g: Profiler2. Cell-cell communication was analyzed by exploring ligand-receptor interactions, particularly between endothelial/Sub-cluster C1 and other cell types, using datasets from established repositories. Systematic analysis of these interactions utilized CellPhoneDB to annotate membrane, secreted, and peripheral proteins under various experimental conditions. Interaction significance was assessed, with p-values < 0.05 indicating significant results. The sequencing and bioinformatics processes were performed by Genechem Co., Ltd. (Shanghai, China). SnRNA-Seq data were deposited in the NIH National Library of Medicine (accession number: PRJNA1180752).

**Golgi-Cox staining**

Golgi-Cox staining was performed to assess changes in the hippocampal (WT, db/db-Veh, and db/db-EVs; four weeks post-injection) synaptic structure as previously described [Zhang, et al. 2020; Wang, et al. 2021]. The experimental brains were removed and stained with Golgi-Cox solution (Table 1), coronal slices (200-μm-thick) including hippocampus (Bregma: 1.3-0.5 mm) were processed and imaged with a confocal microscope (FV3000, Olympus, Japan). 9-10 pyramidal neurons were randomly selected from the experimental groups (n = 3), as pervious documented [Guo, et al. 2019]. A three-dimensional analysis of dendritic morphology was conducted using a computerized tracing system (Neurolucida/Neuroexplorer, MicroBrightField, Inc., USA). The summary analysis of the neuronal data, including total dendritic length, number of dendritic branch points, total dendritic volume, and dendritic complexity index was collected using Neurolucida Explorer (MBF Bioscience, USA).

**Whole-cell patch-clamp recording**

Whole-cell patch-clamp recording was performed 4 weeks after administration, as previously established protocol [Guo, et al. 2018; Wang, et al. 2019a]. Briefly, the experimental mice (WT, db/db-Veh, and db/db-EVs) were anesthetized with isoflurane and transcardially perfused with ice-cold carbogenated (95% O2, 5% CO2) cutting solution (Table 1). Coronal slices containing the hippocampus (300 μm, Bregma: 1.3–0.5 mm) were prepared using a vibrating oscillator (7000 smz-2, Campden Instruments, UK). Sections were placed in a recording tank and continued infusion of artificial cerebrospinal fluid (Table 1) at 25-28°C. Recording micropipettes (BF150-86-7.5, Sutter Instruments, USA) were pulled in a horizontal pipette puller (P-97, Sutter Instruments, USA) with a tip resistance of 3–6 MΩ. Patch pipettes were ﬁlled with the internal solution (Table 1). Currents recordings were made using an Axopatch 700B amplifier (Axon Instruments, Molecular Devices, CA, USA). Signals were filtered at 20 kHz and sampled at 5 kHz with a Digidata 1550B and Clampex 10.7 (Molecular Devices, USA). The data were stored on a computer and analyzed using Clampfit 10.6 and MiniAnal software (Molecular Devices, CA, USA).

**Fluorescence in situ hybridization (FISH)**

FISH was employed to detect specific genetic markers in liver and brain specimens from experimental groups 4 weeks following EV therapy. Tissue sections, each 30 µm thick, were meticulously prepared in accordance with established protocols to ensure precision and reproducibility [Guo, et al. 2019]. Sections underwent digestion with proteinase K (Sigma-Aldrich, USA) to facilitate nucleic acid accessibility, followed by thorough rinsing in PBS to remove residual enzyme activity. Denaturation was performed at 72°C for 5 minutes to separate nucleic acid strands, allowing for optimal probe hybridization. Sections were incubated with fluorescently labeled probes (Sangon Biotech Co.,Ltd., Shanghai, China) targeting PDGFB mRNA in liver macrophages (F4/80) (ThermoFisher Scientific, USA), and TTR mRNA in hippocampal endothelial cells (CD34) (ThermoFisher Scientific, USA). Hybridization proceeded overnight at 37°C in a humidified chamber to allow specific probe binding. Non-specifically bound probes were removed by washing slides with saline-sodium citrate (SSC) buffers of increasing stringency, thus ensuring specificity of hybridization signals. Nuclei were counterstained with 4',6-diamidino-2-phenylindole (DAPI; Biosharp, China) to provide a detailed cellular structure context. Sections were mounted using an antifade mounting medium to preserve fluorescence for subsequent analysis. Fluorescent signals were captured using a confocal microscope (FV3000, Olympus, Japan). The spatial distribution and intensity of PDGFB mRNA expression were quantified using ImageJ Pro Plus V 6.0 software (Bethesda, Maryland, USA).

**miRNA modification and treatment**

Based on miRNA target predictions, miR-31-5p was selected for its ability to target PDGFB, with selection criteria prioritizing total score and energy parameters (Table S8). For in vitro experiments, the miRNA inhibitor, mimic, and negative control (NC) were conjugated with 6-Carboxyfluorescein (FAM) and synthesized by GenePharma (Shanghai, China). These constructs were introduced into MSC-EVs (therapeutic EVs) or HEK-293 cell-derived EVs at a concentration of 100 nM using a Sonic Dismembrator (FB505110, Fisher Scientific, USA) following established protocols [Wang, et al. 2019b]. Primary culture of BMDMs were pre-treated with 10 μg/mL of these miRNA-modified EVs (HEK-EV NC, HEK-EV mimic, EV inhibitor), followed by 0.6 mmol/L PA stimulation for 24 hours. The successful incorporation of miRNA into EVs was confirmed via immunofluorescence, and miRNA expression levels in BMDMs were quantified using quantitative PCR (qPCR). For in vivo studies, miR-31-5p^agomir^ and its corresponding agomir NC were synthesized by Ribobio (Guangzhou, China) (Table 1). The oligonucleotides were prepared in PBS and administered to mice through tail vein injections. The dosing regimen, as recommended by the manufacturer, involved administering 5 nM of the oligonucleotides twice weekly over a three-week period. This treatment commenced three weeks after the initiation of the MCD diet [Zou, et al. 2024].

**Flow cytometry**

MSCs were identified via fluorescence flow cytometry using fluorochrome-conjugated monoclonal antibodies against CD105 (PE), CD90 (PE), and CD73 (FITC) and were assessed to be negative for hematopoietic markers CD45 (FITC) and CD34 (FITC) (Table 1) to confirm their mesenchymal origin [Mushahary, et al. 2018]. BMDMs and RAW 264.7 cells were analyzed using antibody panels with F4/80 (PE-Cy7), CD11b (FITC), CD206 (APC), and CD86 (PerCP-Cy5.5) (Table 1) to delineate macrophage subpopulations and assess polarization states. The staining was performed in accordance with established protocols[Gordon, et al. 2005]. Isotype controls matching each fluorochrome were utilized to ensure specificity. Apoptosis of pericytes was detected using the Annexin V-FITC/PI Apoptosis Kit (Elabscience, Wuhan, China) following manufacturer’s guidelines. Cells were suspended in binding buffer and incubated with Annexin V-FITC and Propidium Iodide (PI) for 15 minutes at room temperature, allowing discrimination between early (Annexin V positive, PI negative) and late apoptotic (Annexin V and PI positive) cells. Fluorescence data acquisition was performed using a flow cytometer (Navios FLOW CYTOMETER, Beckman, USA). Calibration was performed using standard Flow-Check Pro Fluorospheres to ensure optimal machine performance. FlowJo software (version 10, TreeStar, USA) was employed for data analysis, ensuring robust identification and quantification of cellular phenotypes and apoptotic states. Cells were initially gated based on forward and side scatter properties to exclude debris and dead cells, followed by subset identification using the specific markers.

**ELISA**

Cell supernatant, serum, and tissue samples were harvested from the experimental group at the indicated time points. Prior to examinations, samples were centrifuged at 3000 × g for 10 minutes at 4°C to remove debris or particulate matter. Supernatants were subsequently collected and stored at −80°C until further analysis. ELISA kits were employed to quantify the levels of various biomarkers, including tumor necrosis factor-alpha (TNF-α), inducible nitric oxide synthase (iNOS), interleukin 4 (IL-4), interleukin 10 (IL-10), PDGFB, transthyretin (TTR), free thyroxine (FT4), all-trans retinoic acid (ATRA), GDF11, transforming growth factor-beta (TGF-β), interleukin 1 beta (IL-1β), brain-derived neurotrophic factor (BDNF), vascular cell adhesion molecule-1 (VCAM-1), endothelin-1 (ET-1), neuron-specific enolase (NSE), Glucose (Glu), Glycated Hemoglobin A1C (GHbA1C), Insulin, low-density lipoprotein cholesterol (LDL-C), triglycerides (TG), alanine aminotransferase (ALT), and aspartate aminotransferase (AST) among the experimental groups (Table 1). Assays were performed in accordance with the manufacturers' protocols. Absorbance was measured at 450 nm using a FlexStation 3 Microplate Reader (Molecular Devices, USA), and data were processed using SoftMax Pro software (Molecular Devices, USA). Standard curves were generated to determine concentrations, and assay validity was ensured by the inclusion of positive and negative controls. Intra- and inter-assay coefficients of variation (CV) were maintained below 10%.

**Immunofluorescence and Histochemistry**

Cell samples and tissue sections (10-30 μm thickness) were obtained from experimental groups for immunostaining, following established protocols [Xian, et al. 2019; Long, et al. 2017]. The primary antibodies comprised against F4/80, glial fibrillary acidic protein (GFAP), CD9, CD34, PDGFB, GDF11, CD68, alpha-smooth muscle actin (α-SMA), neuronal nuclei (NeuN), microtubule-associated protein 2 (MAP2), and platelet-derived growth factor receptor-beta (PDGFRβ), with detailed specifications available in Table 1. Samples were incubated overnight with primary antibodies, followed by incubation with appropriate secondary antibodies (Table 1). Sections were then mounted, dehydrated, cleared, and cover slipped using Permount™ mounting medium. For imaging, an optical microscope (Leica DMi8, Germany) or a confocal microscope (Olympus FV3000, Japan) was used. From each sample in every group, three random fields were selected at 200× magnification to ensure unbiased representation. 3D reconstruction of vascular makers was performed using Imaris9.0 (Bitplane, Switzerland). Data analysis was conducted using ImageJ Pro Plus V6.0 (Bethesda, Maryland, USA).

**Western blotting**

Cell and tissue samples were collected from experimental groups and prepared as previously described[Yang, et al. 2024]. Samples were lysed in radioimmunoprecipitation assay (RIPA) buffer (Beyotime, China) and protein concentrations were determined using a BCA protein assay kit. Equal amounts of protein were resolved by sodium dodecyl sulfate-polyacrylamide gel electrophoresis (SDS-PAGE) and transferred to polyvinylidene fluoride (PVDF) membranes (331789; Merck Millipore Ltd, Ireland). Primary antibodies against PDGFRβ, phosphorylated PDGFRβ (pPDGFRβ), G-protein signaling 5 (RGS5), GDF11, PDGFB, CD81, CD63, CD9, TSG101, Calnexin, S100β, aquaporin 4 (Aqp4), high mobility group box 1 (HMGB1), phosphorylated small mothers against decapentaplegic 2 (pSMAD2), small mothers against decapentaplegic 2 (SMAD2), Synapsin I (SYN1), p-protein kinase B (p-AKT), AKT, p-nuclear factor kappa-light-chain-enhancer of activated B cells (p-NF-κB/p-p65), p65, VE-Cadherin, Occludin, Connexin 43, GAPDH, and β-actin were used as detailed in Table 1. Membranes were incubated with the appropriate secondary antibodies (Table 1) at room temperature for 1.5 hours. Each sample was analyzed with at least one technical replicate to ensure reliability. Protein bands were visualized using a Bio-Rad imaging system (Bio-Rad, Hercules, CA, USA) and quantified with Image Lab software (Bio-Rad, CA, USA).

**qPCR**

qPCR was conducted to evaluate the expression of miRNAs (miR-31-5p, miR-24-3p) and genes (PDGFB, TTR, Ctla2a) in EVs, cells, or tissues (hippocampus, liver, heart, kidneys) as previously described [Zhao, et al. 2022; Yang, et al. 2024]. Total RNA was isolated using the RNA Easy Fast Tissue/Cell Kit (Qiagen, DE), and RNA integrity and concentration were assessed with a NanoDrop 2000 spectrophotometer (Thermo Fisher Scientific, USA). Primer sequences (Table 1) optimized by Takara Bio Inc. are detailed in Table 1. cDNA was synthesized from 1 µg of total RNA using the High-Capacity cDNA Reverse Transcription Kit (Thermo Fisher Scientific, USA). qPCR was carried out using the TB Green Premix Ex Taq II (Takara, Japan) on a CFX Connect Real-Time PCR System (Bio-Rad, CA, USA). Relative gene expression levels were determined using the 2^-ΔΔCT method, with GAPDH or U6 serving as internal controls. All reactions were performed in triplicate to ensure reproducibility and analyzed with Bio-Rad CFX Manager Software 2.1.

**Statistical analysis**

All statistical analyses were conducted using GraphPad Prism 10.0 (GraphPad Software, USA). Data are presented as mean ± standard error of the mean (SEM). Detailed statistical information, including the statistical tests performed, exact n values, and what n represents, can be found in the figure legends. Data visualization included bar graphs, scatter plots, and line graphs to clearly present the study's findings. The Shapiro-Wilk test was performed to evaluate the normality of data distribution. For comparisons involving multiple groups, one-way ANOVA and repeated measures ANOVA were used, followed by Bonferroni post hoc tests to account for potential Type I errors. For comparisons between two groups, an unpaired two-tailed Student's t-test was utilized for normally distributed data, while the Mann-Whitney U test was applied for non-normally distributed data. Statistical significance was defined as P < 0.05.

**References**

Zhang X., L. Fan, J. Wu, et al. 2019."Macrophage p38α promotes nutritional steatohepatitis through M1 polarization." Journal of hepatology 71, no. 1: 163-174.

Kojima H., M. Fujimiya, K. Matsumura, et al. 2003."NeuroD-betacellulin gene therapy induces islet neogenesis in the liver and reverses diabetes in mice." Nature medicine 9, no. 5: 596-603.

Xian P., Y. Hei, R. Wang, et al. 2019."Mesenchymal stem cell-derived exosomes as a nanotherapeutic agent for amelioration of inflammation-induced astrocyte alterations in mice." Theranostics 9, no. 20: 5956-5975.

Wang T., Z. Jian, A. Baskys, et al. 2020."MSC-derived exosomes protect against oxidative stress-induced skin injury via adaptive regulation of the NRF2 defense system." Biomaterials 257, no. 120264.

Kazankov K., S. M. D. Jørgensen, K. L. Thomsen, et al. 2019."The role of macrophages in nonalcoholic fatty liver disease and nonalcoholic steatohepatitis." Nature reviews. Gastroenterology & hepatology 16, no. 3: 145-159.

Ying W., P. S. Cheruku, F. W. Bazer, S. H. Safe, B. Zhou 2013."Investigation of macrophage polarization using bone marrow derived macrophages." Journal of visualized experiments, no. 76.

Ni X. X., P. X. Ji, Y. X. Chen, et al. 2022."Regulation of the macrophage-hepatic stellate cell interaction by targeting macrophage peroxisome proliferator-activated receptor gamma to prevent non-alcoholic steatohepatitis progression in mice." Liver international 42, no. 12: 2696-2712.

Luo Q., P. Xian, T. Wang, et al. 2021."Antioxidant activity of mesenchymal stem cell-derived extracellular vesicles restores hippocampal neurons following seizure damage." Theranostics 11, no. 12: 5986-6005.

Moigneu C., S. Abdellaoui, M. Ramos-Brossier, et al. 2023."Systemic GDF11 attenuates depression-like phenotype in aged mice via stimulation of neuronal autophagy." Nature aging 3, no. 2: 213-228.

Suragani R. N., S. M. Cadena, S. M. Cawley, et al. 2014."Transforming growth factor-β superfamily ligand trap ACE-536 corrects anemia by promoting late-stage erythropoiesis." Nature medicine 20, no. 4: 408-414.

Zhao T., G. Zhong, Y. Wang, et al. 2024."Pregnane X Receptor Activation in Liver Macrophages Protects against Endotoxin-Induced Liver Injury." Advanced Science 11, no. 19: e2308771.

Gu M., M. Donato, M. Guo, et al. 2021."iPSC-endothelial cell phenotypic drug screening and in silico analyses identify tyrphostin-AG1296 for pulmonary arterial hypertension." Science translational medicine 13, no. 592: eaba6480.

Kornilov R., M. Puhka, B. Mannerstrom, et al. 2018."Efficient ultrafiltration-based protocol to deplete extracellular vesicles from fetal bovine serum." Journal of extracellular vesicles 7, no. 1: 1422674.

Zhao R., L. Wang, T. Wang, et al. 2022."Inhalation of MSC-EVs is a noninvasive strategy for ameliorating acute lung injury." Journal of controlled release 345, no. 214-230.

Thery C., S. Amigorena, G. Raposo, A. Clayton 2006."Isolation and characterization of exosomes from cell culture supernatants and biological fluids." Current protocols in cell biology Chapter 3, no. Unit 3 22.

Crewe C., N. Joffin, J. M. Rutkowski, et al. 2018."An Endothelial-to-Adipocyte Extracellular Vesicle Axis Governed by Metabolic State." Cell 175, no. 3: 695-708.e613.

Thery C., K. W. Witwer, E. Aikawa, et al. 2018."Minimal information for studies of extracellular vesicles 2018 (MISEV2018): a position statement of the International Society for Extracellular Vesicles and update of the MISEV2014 guidelines." Journal of extracellular vesicles 7, no. 1: 1535750.

Lu Y., Y. Zhang, Z. Lou, et al. 2024."Metagenomic next-generation sequencing of cell-free DNA for the identification of viruses causing central nervous system infections." Microbiology spectrum 12, no. 1: e0226423.

Witwer K. W., B. W. M. Van Balkom, S. Bruno, et al. 2019."Defining mesenchymal stromal cell (MSC)-derived small extracellular vesicles for therapeutic applications." Journal of extracellular vesicles 8, no. 1: 1609206.

Chung Y. H., Y. P. Ho, S. S. Farn, et al. 2024."In vivo SPECT imaging of Tc-99 m radiolabeled exosomes from human umbilical-cord derived mesenchymal stem cells in small animals." Biomedical journal 47, no. 5: 100721.

Yang Y., L. Gao, J. Xi, et al. 2024."Mesenchymal stem cell-derived extracellular vesicles mitigate neuronal damage from intracerebral hemorrhage by modulating ferroptosis." Stem cell research & therapy 15, no. 1: 255.

Long Q., D. Upadhya, B. Hattiangady, et al. 2017."Intranasal MSC-derived A1-exosomes ease inflammation, and prevent abnormal neurogenesis and memory dysfunction after status epilepticus." Proceedings of the National Academy of Sciences of the United States of America 114, no. 17: E3536-E3545.

Lu Y. T., T. Y. Chen, H. H. Lin, et al. 2025."Small Extracellular Vesicles Engineered Using Click Chemistry to Express Chimeric Antigen Receptors Show Enhanced Efficacy in Acute Liver Failure." Journal of extracellular vesicles 14, no. 2: e70044.

Cheng K., R. Kalluri 2023."Guidelines for clinical translation and commercialization of extracellular vesicles and exosomes based therapeutics." Extracellular Vesicle 2, no. 100029.

Yang L., L. Tian, Z. Zhang, et al. 2020."Cannabinoid Receptor 1/miR-30b-5p Axis Governs Macrophage NLRP3 Expression and Inflammasome Activation in Liver Inflammatory Disease." Molecular therapy. Nucleic acids 20, no. 725-738.

Zou Z., X. Liu, J. Yu, et al. 2024."Nuclear miR-204-3p mitigates metabolic dysfunction-associated steatotic liver disease in mice." Journal of hepatology 80, no. 6: 834-845.

Uhl P., G. Fricker, U. Haberkorn, W. Mier 2015."Radionuclides in drug development." Drug discovery today 20, no. 2: 198-208.

Liu H., X. Huang, J. Xu, et al. 2021."Dissection of the relationship between anxiety and stereotyped self-grooming using the Shank3B mutant autistic model, acute stress model and chronic pain model." Neurobiology of stress 15, no. 100417.

Zhang X. Q., L. Xu, Y. Ling, et al. 2021."Diminished excitatory synaptic transmission correlates with impaired spatial working memory in neurodevelopmental rodent models of schizophrenia." Pharmacology, biochemistry, and behavior 202, no. 173103.

Nelson E. D., K. R. Maynard, K. R. Nicholas, et al. 2023."Activity-regulated gene expression across cell types of the mouse hippocampus." Hippocampus 33, no. 9: 1009-1027.

Wolf F. A., P. Angerer, F. J. Theis 2018."SCANPY: large-scale single-cell gene expression data analysis." Genome biology 19, no. 1: 15.

Wolf F. A., F. K. Hamey, M. Plass, et al. 2019."PAGA: graph abstraction reconciles clustering with trajectory inference through a topology preserving map of single cells." Genome biology 20, no. 1: 59.

Zhang Y., Z. Xiao, Z. He, et al. 2020."Dendritic complexity change in the triple transgenic mouse model of Alzheimer's disease." PeerJ 8, no. e8178.

Wang H., Y. Liu, J. Li, et al. 2021."Tail-vein injection of MSC-derived small extracellular vesicles facilitates the restoration of hippocampal neuronal morphology and function in APP / PS1 mice." Cell death discovery 7, no. 1: 230.

Guo B., J. Chen, Q. Chen, et al. 2019."Anterior cingulate cortex dysfunction underlies social deficits in Shank3 mutant mice." Nature neuroscience 22, no. 8: 1223-1234.

Guo B., J. Wang, H. Yao, et al. 2018."Chronic Inflammatory Pain Impairs mGluR5-Mediated Depolarization-Induced Suppression of Excitation in the Anterior Cingulate Cortex." Cerebral cortex (New York, N.Y. : 1991) 28, no. 6: 2118-2130.

Wang M., X. Liu, Y. Hou, et al. 2019a."Decrease of GSK-3β Activity in the Anterior Cingulate Cortex of Shank3b (-/-) Mice Contributes to Synaptic and Social Deficiency." Frontiers in cellular neuroscience 13, no. 447.

Wang P., H. Wang, Q. Huang, et al. 2019b."Exosomes from M1-Polarized Macrophages Enhance Paclitaxel Antitumor Activity by Activating Macrophages-Mediated Inflammation." Theranostics 9, no. 6: 1714-1727.

Mushahary D., A. Spittler, C. Kasper, V. Weber, V. Charwat 2018."Isolation, cultivation, and characterization of human mesenchymal stem cells." Cytometry 93, no. 1: 19-31.

Gordon S., P. R. Taylor 2005."Monocyte and macrophage heterogeneity." Nature reviews. Immunology 5, no. 12: 953-964.

**SI Figures**


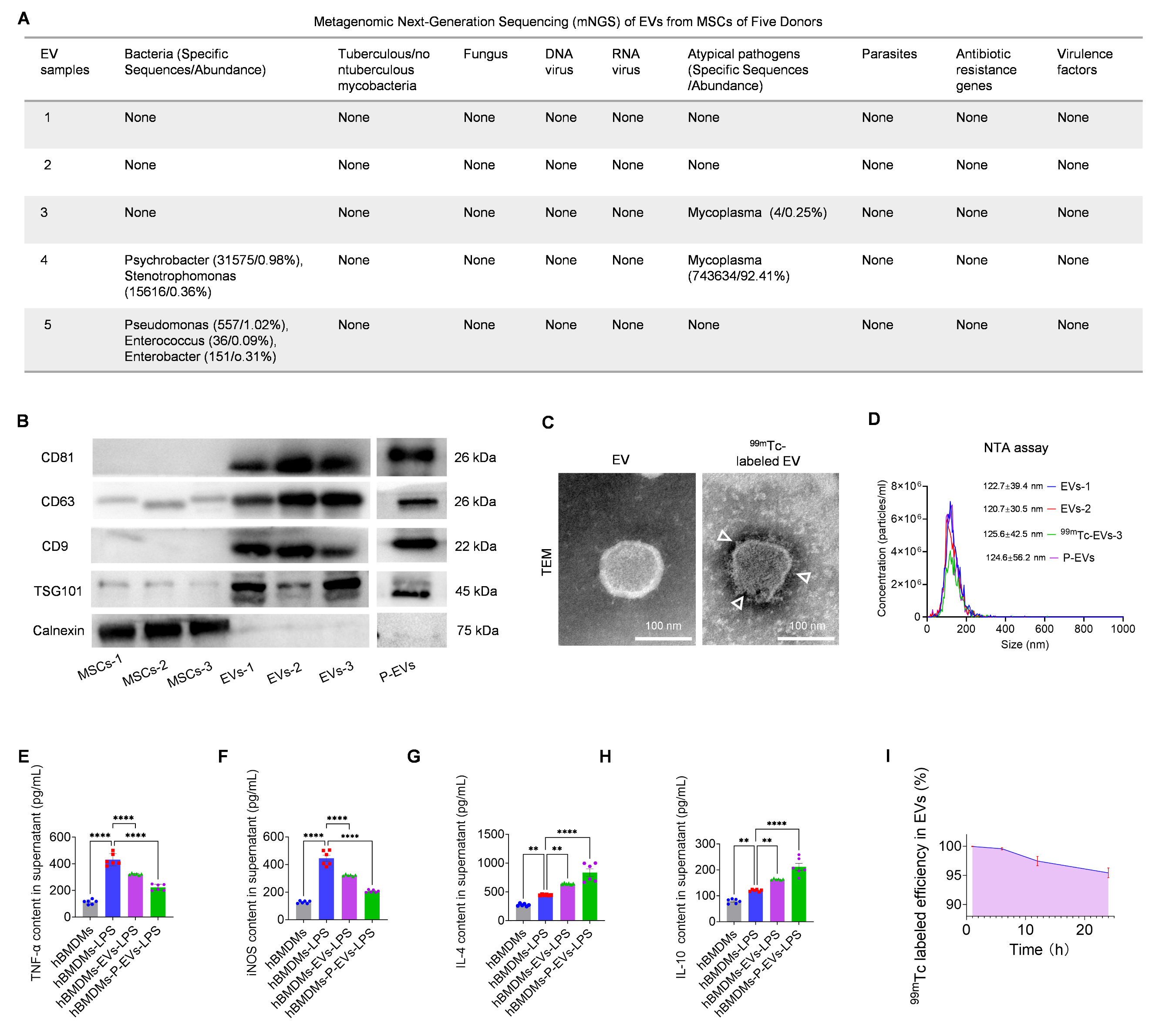


**Figure S1. Therapeutic EVs identification and labeling.**

(A) Metagenomic next-generation sequencing (mNGS) analysis was performed to assess microbial contamination across extracellular vesicle (EV) samples derived from five mesenchymal stromal cell (MSC) donors. Bacterial sequences were detected in EV-3 (Mycoplasma, 4.0%) and EV-5 (Mycoplasma, 92.41%), alongside other minor bacterial contaminations in EV-4 and EV-5, leading to the selection of EV-1, EV-2, and EV-3 for further experiments (minimal/no contamination). mNGS data have been deposited in the NIH Sequence Read Archive (SRA) under accession number PRJNA1181240. (B) Western blot analysis of EV-specific markers (CD81, CD63, CD9, TSG101) confirmed identity across EV-1, EV-2, EV-3, and purified EVs (P-EVs), while the intracellular marker Calnexin was absent, ensuring purity. Whole-cell lysates of MSCs served as controls. (C) Transmission electron microscopy (TEM) revealed the characteristic morphology of EVs, with arrowheads indicating the integration of ^99m^Tc on EV membranes. Scale bars: 100 nm. (D) Nanoparticle tracking analysis (NTA) confirmed consistent size distributions and particle concentrations across EV-1, EV-2, ^99m^Tc-labled EVs (EV-3), and P-EVs with mean diameters of 122.7 ± 39.4 nm, 120.7 ± 30.5 nm, 125.6 ± 42.5 nm, and 124.6 ± 56.2, respectively. (E–H) Cytokine profiling in human bone marrow-derived macrophages (hBMDMs) evaluated the immunomodulatory effects of EVs under baseline (hBMDMs), lipopolysaccharide (LPS) stimulation (hBMDMs-LPS), and EVs or P-EVs preconditioning followed by LPS challenge (hBMDMs-EVs-LPS / hBMDMs-P-EVs-LPS). ELISA quantification demonstrated that, both EVs and P-EVs significantly suppressed pro-inflammatory markers TNF-α (E) and iNOS (F), while enhancing anti-inflammatory cytokines IL-4 (G) and IL-10 (H). (I) Efficiency of ^99m^Tc labeling (> 95%) in EVs over 0-24 hours (h) (n = 5 independent samples per time point). All data are represented as mean ± SEM. “n” denotes the number of biological and technical replicates (E–H), or biological replicates (I). Statistical analysis was performed using one-way ANOVA with Bonferroni post hoc tests (E-H). Significance levels: ***p* < 0.01; ****p* < 0.001; *****p* < 0.0001.


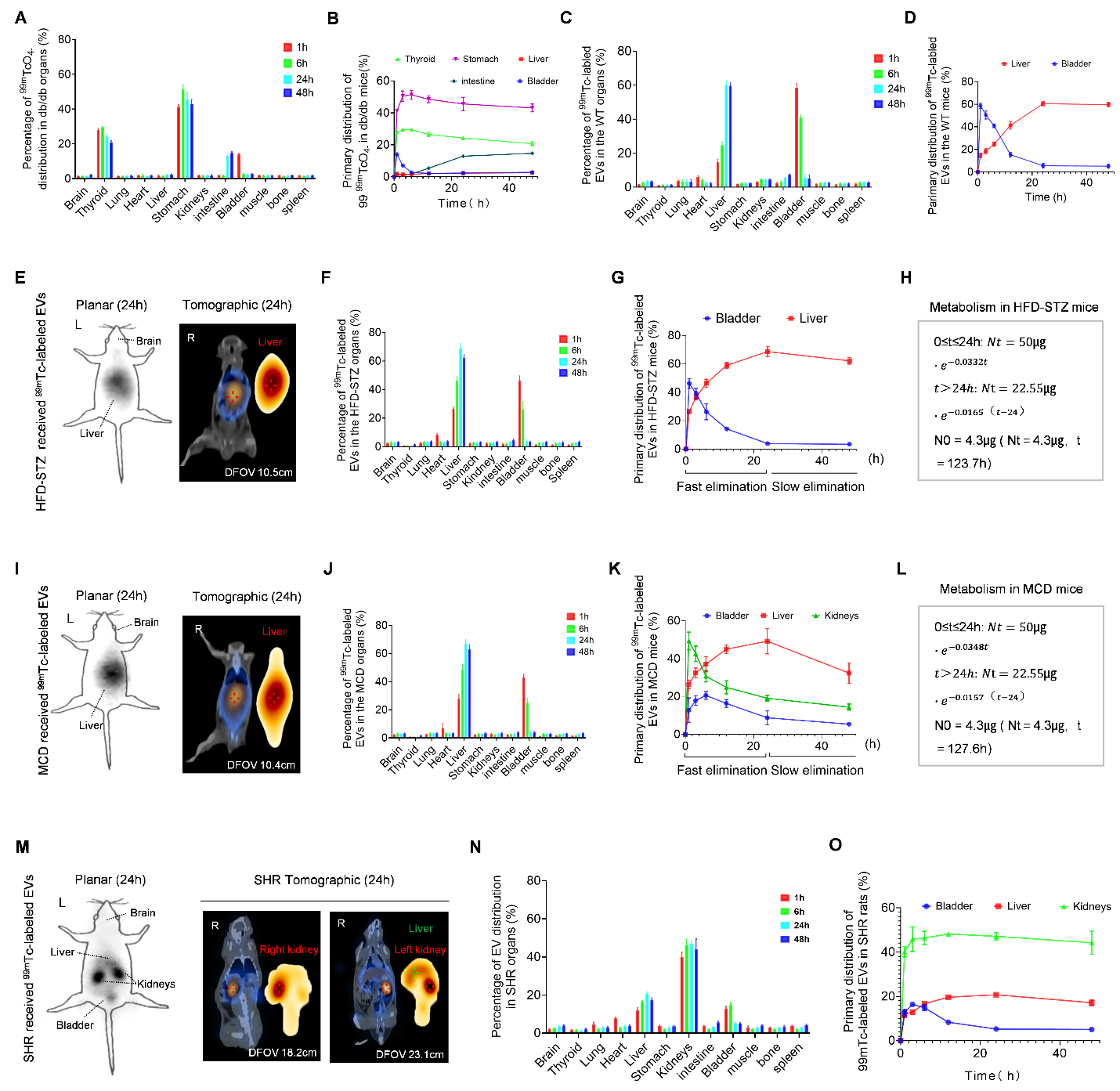


**Figure S2. SPECT/CT imaging and analysis of ^99m^Tc-labeled EVs across different models.**

(A, B) Distribution of ⁹⁹ᵐTc pertechnetate ion (⁹⁹ᵐTcO₄⁻) in db/db mice at 1-, 6-, 24-, and 48-hours post-injection (n = 3 per time point). (A) Significant accumulation was observed in the thyroid and stomach, with minimal liver distribution. (B) Thyroid and stomach retention remained consistent over time. (C, D) Distribution of ⁹⁹ᵐTc-labeled EVs in WT mice at 1-, 6-, 24-, and 48-hours post-injection (n = 3 per time point). (C) EVs predominantly accumulated in the liver and bladder. (D) Liver retention was sustained, while bladder accumulation decreased after 24 hours. (E, F) Imaging and distribution of ⁹⁹ᵐTc-labeled EVs in HFD-STZ mice at 24 hours post-injection (DFOV 10.5 cm, n = 5). (E) Imaging showed strong liver and bladder retention. (F) Quantitative data confirmed liver and bladder as the primary retention sites.
(G, H) Liver and bladder distribution dynamics of ⁹⁹ᵐTc-labeled EVs in HFD-STZ mice over time (n = 5). (G) Bladder elimination was rapid, while liver retention was sustained. (H) Metabolic analysis revealed a half-life (t½) of 123.7 hours for the slow elimination phase.
(I, J) Imaging and distribution of ⁹⁹ᵐTc-labeled EVs in MCD mice at 24 hours post-injection (DFOV 10.4 cm, n = 5). (I) Imaging showed significant liver and bladder retention. (J) Liver retention remained consistent across time points. (K, L) Liver, bladder, and kidney distribution of ⁹⁹ᵐTc-labeled EVs in MCD mice over time (n = 5). (K) Liver retention remained high, while bladder and kidney levels decreased after 24 hours. (L) Metabolic analysis showed a half-life (t½) of 127.6 hours for the slow elimination phase. (M, N) Imaging and distribution of ⁹⁹ᵐTc-labeled EVs in SHR rats at 24 hours post-injection (DFOV 18.2 cm and 23.1 cm, n = 3). (M) Imaging revealed predominant retention in the liver, bladder, and kidneys. (N) Liver retention was the highest, with bladder and kidney levels decreasing over time. All data are presented as mean ± SEM. “n” represents biological replicates.


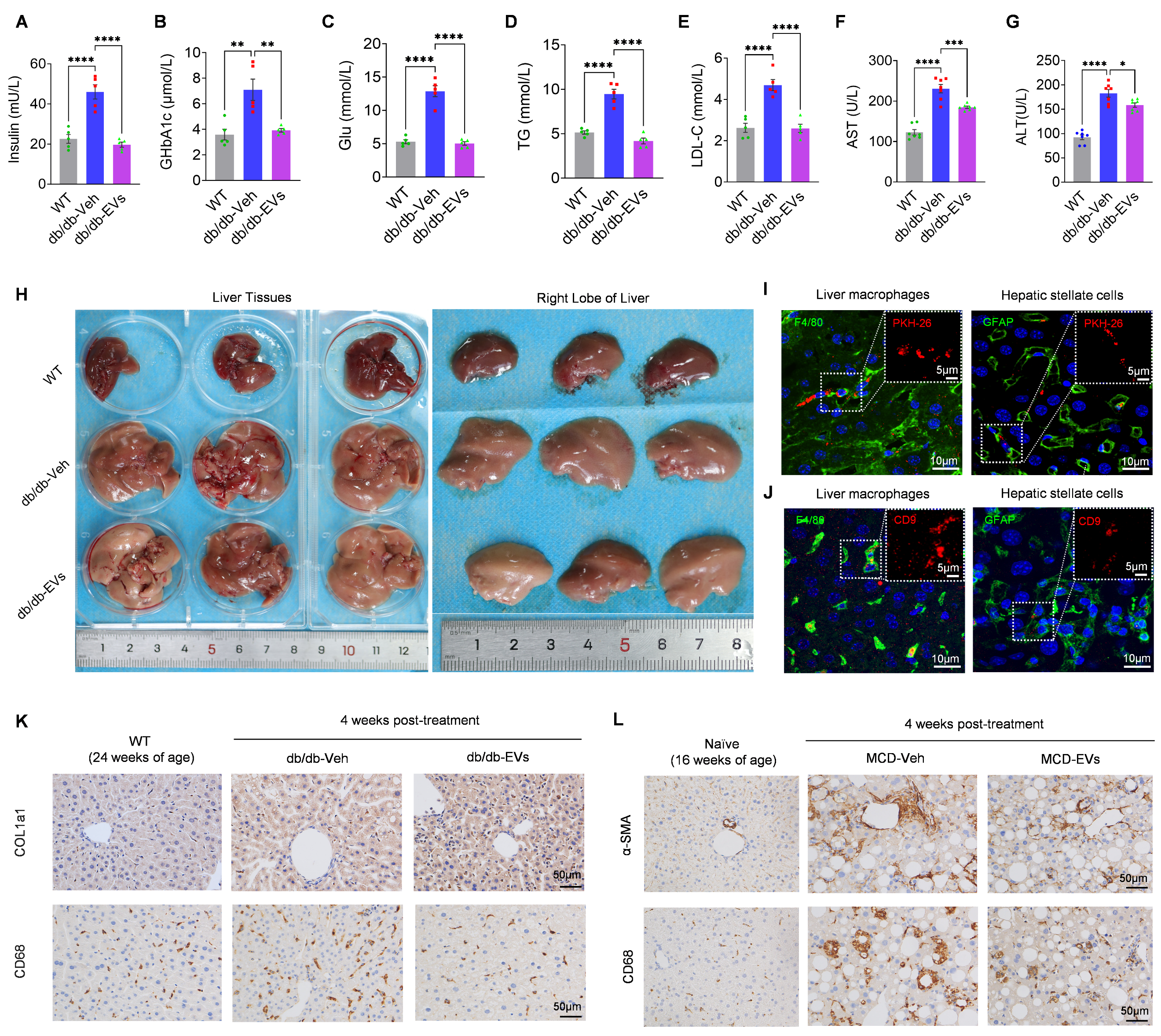


**Figure S3. Metabolic and NAFLD evaluation in recipient mice after EV infusion.**

(A–G) Serum insulin (A), glycosylated hemoglobin (HbA1c, B), glucose (Glu, C), triglyceride (TG, D), low-density lipoprotein cholesterol (LDL-C, E), aspartate aminotransferase (AST, F), and alanine aminotransferase (ALT, G) levels in limosis of WT, db/db-Veh, and db/db-EVs groups (n = 7). EV treatment significantly reduced these db/db-associated metabolic risk factors compared to vehicle controls. (H) Representative macroscopic images of liver tissues from WT, db/db-Veh, and db/db-EVs groups, showing improved liver morphology in db/db-EVs livers compared to db/db-Veh. (I, J) PKH26-labeled EVs (red, I) and EV marker CD9 (red, J) co-localized with liver macrophages (F4/80, green) and hepatic stellate cells (GFAP, green) at 20 hours post-injection, the peak aggregation time point (n = 3). These images show more EVs integrated to macrophages. Scale bars: 10 μm (overview), 5 μm (insets). (K) Immunohistochemistry of liver sections from the experimental groups at 4 weeks post-treatment. Staining for COL1a1 and CD68 revealed reduced fibrosis and macrophage activation in db/db-EVs livers compared to db/db-Veh. Scale bars: 50 μm. (L) Immunohistochemical staining for α-SMA and CD68 in liver sections from MCD mice 4 weeks post-treatment. EV-treated MCD mice exhibited reduced fibrosis and inflammation compared to vehicle-treated controls. Scale bars: 50 μm. Data are presented as mean ± SEM. “n” represents biological replicates. Statistical analysis was performed using one-way ANOVA with Bonferroni post hoc tests (A–G). Significance levels: **p* < 0.05; ** *p* < 0.05; *** *p* < 0.001; **** *p* < 0.0001.


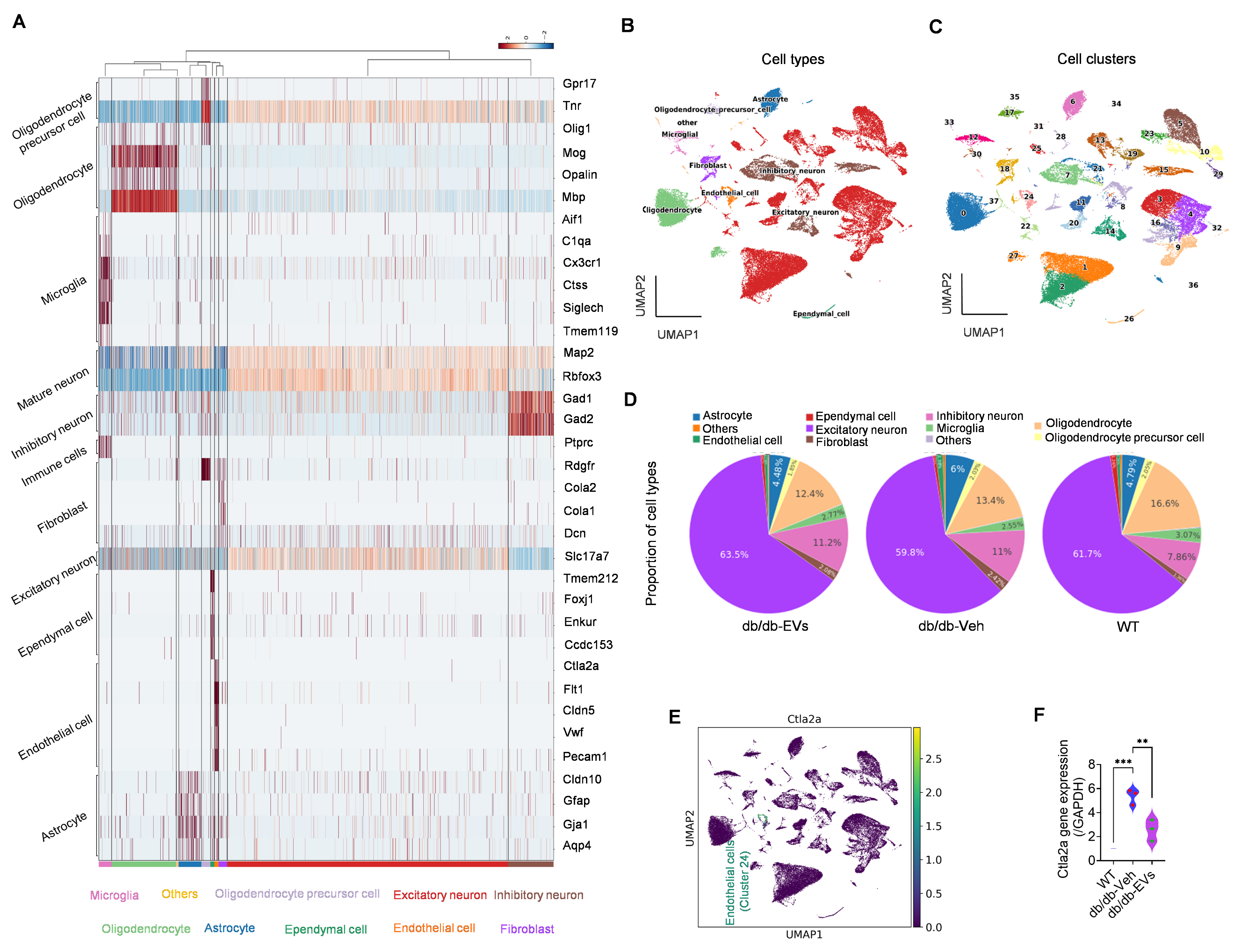


**Figure S4. SnRNA-Seq profiling and *Ctla2a* verification in recipient hippocampus.**

(A) Heatmap of canonical marker genes across hippocampal cell types, including neurons, astrocytes, microglia, oligodendrocytes, and others, demonstrating clear cell type-specific expression patterns. (B) UMAP visualization displaying hippocampal cell types in distinct clusters, identified based on marker gene expressions, each type represented by a different color. (C) UMAP distribution of 38 unique cell clusters (0–37) identified through differentially expressed genes (DEGs). (D) Pie charts comparing relative proportions of hippocampal cell types in WT, db/db-Veh, and db/db-EVs groups. EV treatment altered the proportions of astrocytes, excitatory neurons, and other cell types compared to db/db-Veh mice. (E) UMAP of Cluster 24 with mark gene *Ctla2a*. (F) Violin plot of *Ctla2a* expression in WT, db/db-Veh, and db/db-EVs groups (n = 3), showing EV-induced downregulation compared to vehicle controls. Data are presented as mean ± SEM. “n” represents biological replicates. Statistical analysis was performed using one-way ANOVA with Bonferroni post hoc test (F). Significance levels: ** *p* < 0.01, *** *p* < 0.001.


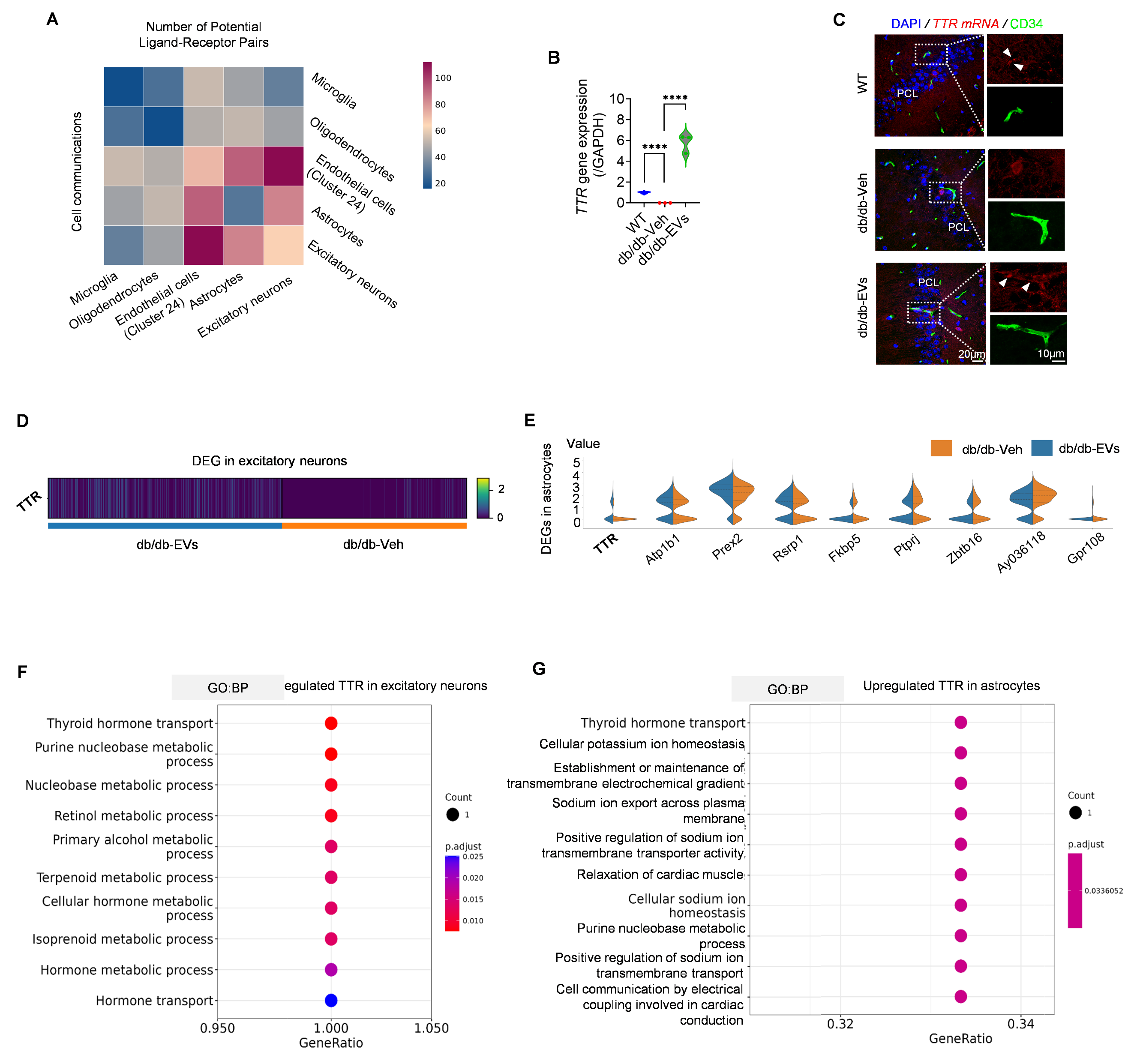


**Figure S5. Cell communication and DEG analysis of the experimental hippocampus.**

(A) Cell communication analysis (CellPhoneDB) illustrating the ligand-receptor interactions among endothelial cells (Cluster 24), astrocytes, excitatory neurons, microglia, and oligodendrocytes in the experimental groups. (B) qPCR results confirming significantly increased expression of *TTR* in hippocampal tissues of EVs-treated db/db mice compared to db/db-Veh controls (n = 3, biological replicates). (C) Fluorescence in situ hybridization (FISH) images showing *TTR mRNA* (red) colocalized with CD34 (green), an endothelial cell marker, in the hippocampal pyramidal cell layer (PCL). White arrows indicate changes in *TTR mRNA* in endothelial cells (CD34+ cells). Scale bars: 20 µm (left), 10 µm (right). (D) Heatmap of DEGs in excitatory neurons showing higher expression of *TTR* in db/db-EVs compared to db/db-Veh mice. (E) Violin plots visualizing expression levels of DEGs in astrocytes, including *TTR* upregulation and other genes across db/db-Veh and db/db-EVs groups. (F, G) Gene Ontology (GO): Biological Process (BP) enrichment analyses of pathways associated with upregulated *TTR* in excitatory neurons (F) and astrocytes (G). Enriched pathways include thyroid hormone transport and retinol metabolism, indicating potential restoration of functional processes. Dot size represents the number of genes involved, and color represents the adjusted P-value. DEGs were identified using Seurat's FindAllMarkers function with thresholds of absolute fold change ≥ 1.2 and adjusted P-value ≤ 0.05. Functional enrichment analysis of DEGs was performed using g: Profiler2. Statistical validation utilized the Wilcoxon test.


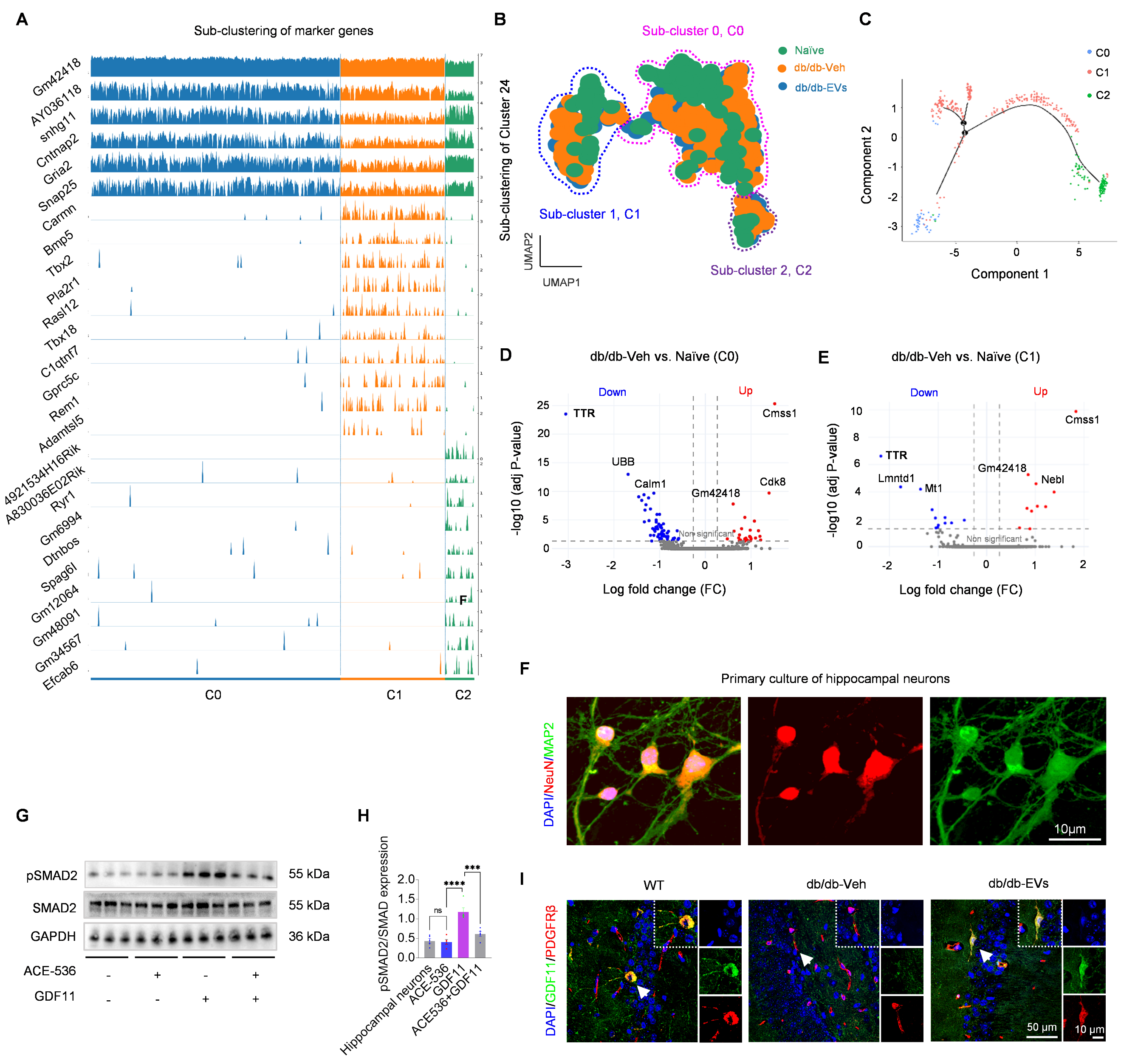


**Figure S6. Re-clustering of Cluster 24 and validation of GDF11 expression.**

(A) Heatmap showing the expression of highly variable genes across sub-clusters C0, C1, and C2 within Cluster 24, generated using the pp.neighbors function. Distinct transcriptional profiles highlight the heterogeneity within Cluster 24. (B) UMAP visualization of sub-clusters C0 (orange), C1 (blue), and C2 (purple) within Cluster 24, demonstrating clear separation of cells based on gene expression patterns. (C) Pseudotime trajectory analysis of Cluster 24 cells, revealing a gradient of gene expression changes that define the relationships among sub-clusters C0, C1, and C2, supporting their transcriptional divergence. (D, E) Volcano plots illustrating differentially expressed genes (DEGs) between the vehicle-treated (db/db-Veh) and WT groups in sub-clusters C0 (D) and C1 (E). DEGs were not shown in C2. Significant DEGs (adjusted p-value ≤ 0.05, |fold change| ≥ 1.2) are highlighted in red (upregulated) and blue (downregulated). The top three regulated genes are labeled. DEGs were identified using Seurat’s FindAllMarkers with the Wilcoxon test. (F) Immunofluorescence images of primary hippocampal neurons stained for NeuN (red), microtubule-associated protein 2 (MAP2, green), and 4',6-diamidino-2-phenylindole (DAPI, blue), confirming neuronal identity. Scale bar, 10 μm. (G) Western blot analysis of pSMAD2 and SMAD2 in hippocampal neurons treated with ACE-536 and/or recombinant GDF11 protein. GAPDH was used as the loading control. (H) Bar graph quantifying pSMAD2/SMAD2 protein levels in hippocampal neurons treated with recombinant GDF11 and/or ACE-536 (an inhibitor of SMAD2/3), illustrating the cascade of GDF11-TGFβ receptor signaling. (I) Representative immunofluorescence images of hippocampal CA1 tissues (WT, db/db-Veh, and db/db-EVs) stained for DAPI (blue), GDF11 (green), and PDGFRβ (red). Increased GDF11 expression is observed in pericytes of EVs-treated db/db mice (n = 4). Scale bar, 50 μm. Insets show higher magnification views; scale bar, 10 μm. Data are presented as mean ± SEM. “n” represents technical replicates. Statistical significance was determined using one-way ANOVA with Bonferroni post hoc test (H). Significance levels: ns, *p* > 0.05; *** *p* < 0.001; **** *p* < 0.0001.


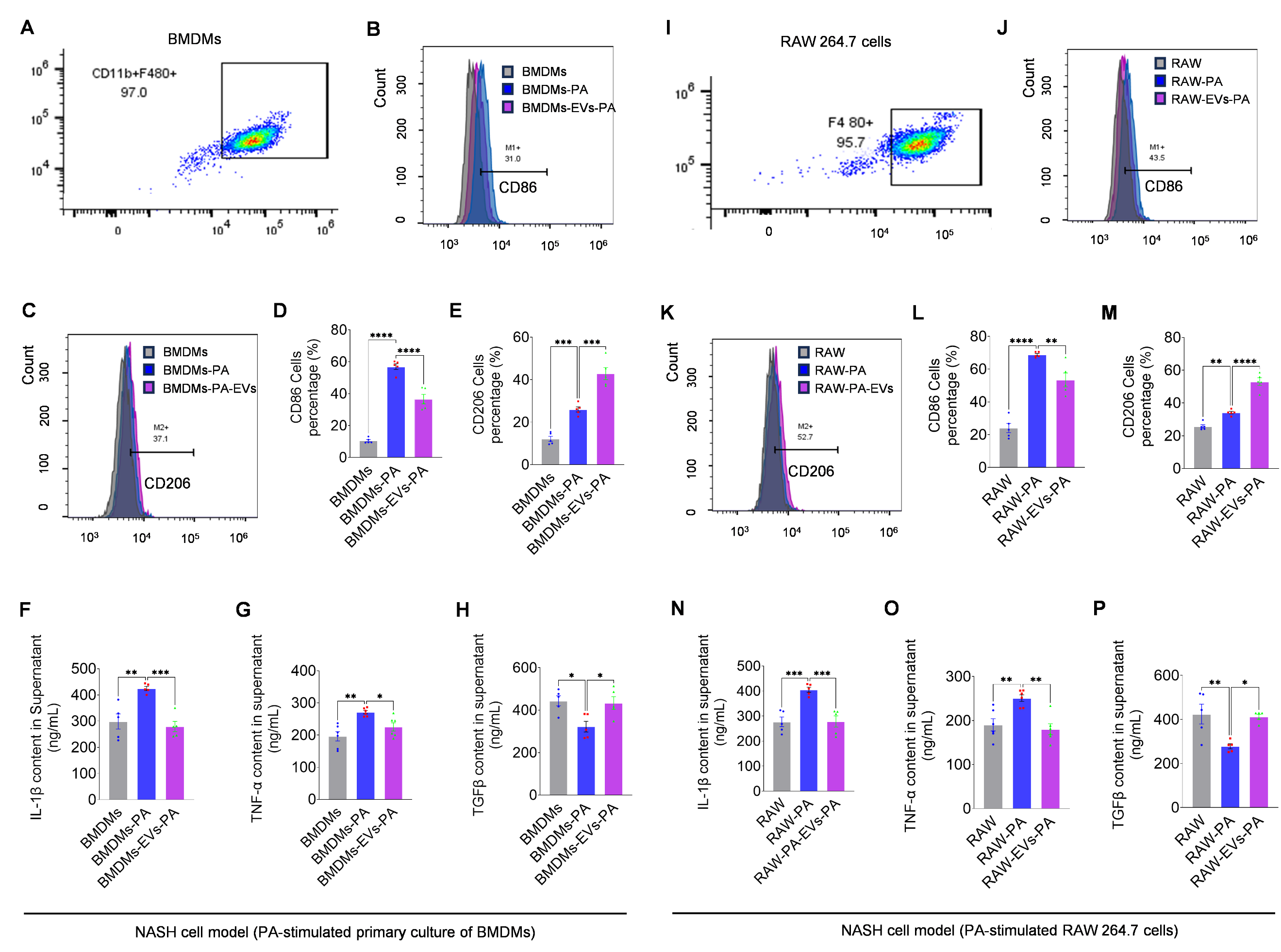


**Figure S7. Immunomodulatory role of EVs on PA-stimulated cell models.**

(A) Flow cytometry analysis displaying the proportion of CD11b⁺F4/80⁺ cells in primary bone marrow-derived macrophage (BMDM) cultures, with over 95% expression observed across at least five cohorts. (B, C) Histograms showing CD86 (M1 marker, B) and CD206 (M2 marker, C) expression in primary BMDMs. Groups include untreated BMDMs, PA-stimulated BMDMs (BMDMs-PA), and BMDMs preconditioned with EVs followed by PA stimulation (BMDMs-EVs-PA). (D, E) Quantification of CD86⁺ (D) and CD206⁺ (E) cells as a percentage of total BMDMs across the three experimental groups (n = 5). (F–H) ELISA measurements of cytokine levels in the supernatant of primary BMDMs cultures. Shown are levels of IL-1β (M1-related, F), TNF-α (M1-related, G), and TGF-β (M2-related, H) across above indicated groups (n = 5).
(I) Flow cytometry analysis showing the percentage of F4/80⁺ cells in RAW 264.7 macrophages, with over 95% expression observed across five cohorts. (J, K) Histograms displaying CD86⁺ (M1 marker, J) and CD206⁺ (M2 marker, K) expression in RAW 264.7 macrophages (RAW) across untreated RAW, PA-stimulated RAW (RAW-PA), and RAW preconditioned with EVs followed by PA stimulation (RAW-EVs-PA). (L, M) Quantification of CD86⁺ (L) and CD206⁺ (M) cells as a percentage of total cells for each indicated group (n = 5). (N–P) ELISA measurements of cytokine levels in the supernatant of RAW 264.7 macrophage cultures. Shown are levels of IL-1β (M1-related, N), TNF-α (M1-related, O), and TGF-β (M2-related, P) across the indicated groups (n = 5). Data are presented as mean ± SEM. “n” represents experimental replicates. Group comparisons were performed using one-way ANOVA with Bonferroni post hoc tests (D-H, L-P). Significance: * *p* < 0.05; ** *p* < 0.01; *** *p* < 0.001; **** *p* < 0.0001.


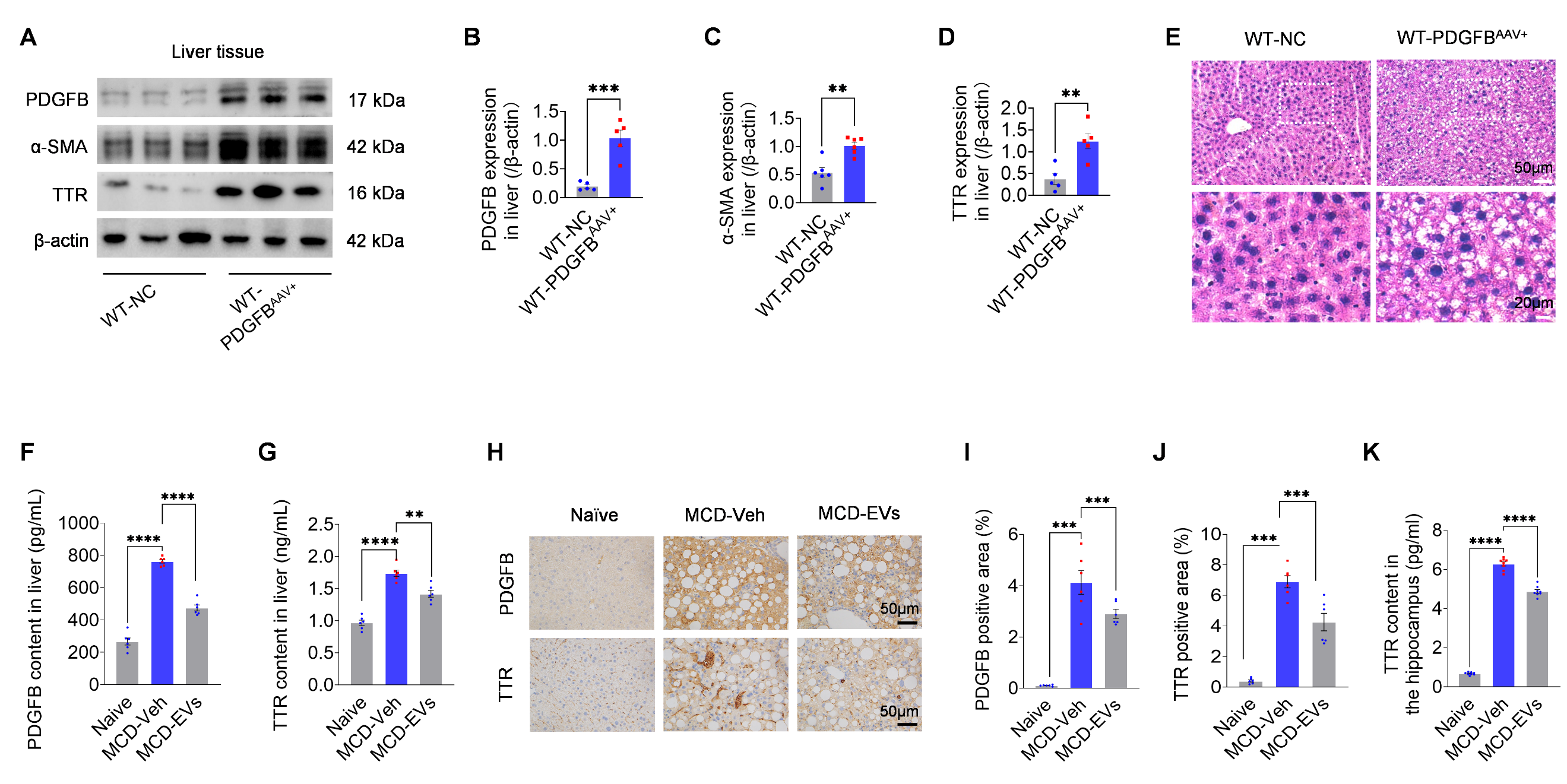


**Figure S8. Association of PDGFB and TTR in recipient mice.**

(A) Western blot analysis of liver tissues from WT mice treated with AAV8-NC (WT-NC) or AAV8-GP-PDGFB-upregulated (WT-PDGFB^AAV+^) injections. (B–D) Protein quantification shows an increase of PDGFB (B), α-SMA (C), and TTR (D) expression in the WT-PDGFB^AAV+^ groups compared to NC group (n = 5). (E) H&E staining of liver sections in WT-NC and WT-PDGFB^AAV+^ groups. WT-PDGFB^AAV+^ sections display histological worsening, marked with ballooning degeneration compared to WT-NC. Scale bars: 20 μm (down panels) and 50 μm (up panels). (F, G) ELISA analysis of liver PDGFB (F) and TTR (G) levels in Naïve, MCD vehicle (MCD-Veh), and MCD received EV infusion (MCD-EVs) groups. EV intervention significantly reduced liver PDGFB levels and TTR synthesis in MCD mice (n = 6). (H–J) Immunohistochemical staining showing PDGFB and TTR expression in liver sections from Naïve, MCD-Veh, and MCD-EVs groups (H). Quantified data further confirm decreased PDGFB (I) and TTR (J) expression in the EV-treated groups compared to vehicle controls (n = 6). Scale bars: 50 μm. (I) ELISA analysis of TTR content in the hippocampus across Naïve, MCD-Veh, and MCD-EVs groups. TTR levels were significantly reduced in the MCD-EVs group compared to the MCD-Veh group (n = 8). Data are presented as mean ± SEM. “n” represents biological replicates. Statistical comparisons were performed using one-way ANOVA with Bonferroni post hoc (F, G, I–K) or unpaired two-tailed Student’s *t*-test where appropriate (B–D). Significance: ***p* < 0.01; *** *p* < 0.001; **** *p* < 0.0001.


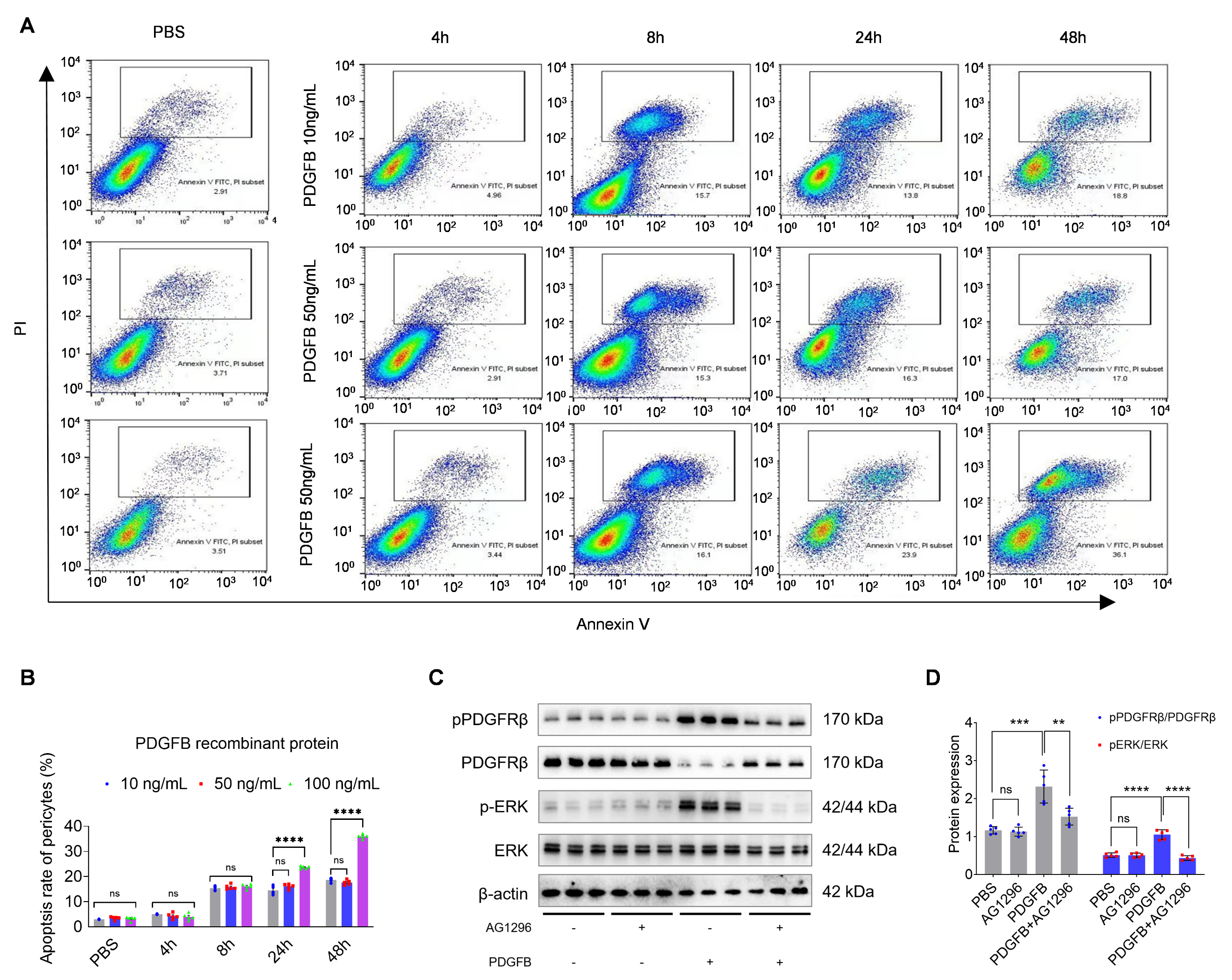


**Figure S9. Regulatory role of PDGFB on pericyte activity.**

(A) Flow cytometry analysis of pericyte apoptosis after treatment with recombinant PDGFB (10, 50, or 100 ng/mL) for 4, 8, 24, and 48 hours (h). Apoptotic cells (Annexin V+ and/or PI+) are highlighted and compared to PBS-treated controls. (B) Quantification of apoptosis rates shows a dose- and time-dependent increase in pericyte apoptosis with PDGFB treatment compared to PBS controls (n = 5). (C) Western blot analysis of PDGFRβ and downstream extracellular signal-regulated kinase (ERK) signaling in pericytes treated with 20 ng/mL PDGFB and/or 10 μM AG1296 (a PDGFRβ inhibitor) for 24h. Phosphorylated PDGFRβ (pPDGFRβ) and ERK (pERK) levels are shown along with total PDGFRβ, ERK, and β-actin controls. (D) Quantification of protein expression (pPDGFRβ/PDGFRβ and pERK/ERK) confirms that AG1296 inhibits PDGFB-induced activation of PDGFRβ and ERK signaling (n = 5). Data are mean ± SEM. “n” denotes the number of experimental replicates (B) and technical replicates (D). Statistical significance was determined by two-way ANOVA for (B) and one-way ANOVA with Bonferroni post hoc test for (D). Significance levels: ns, *p* > 0.05; ** *p* < 0.01; *** *p* < 0.001; **** *p* < 0.0001.


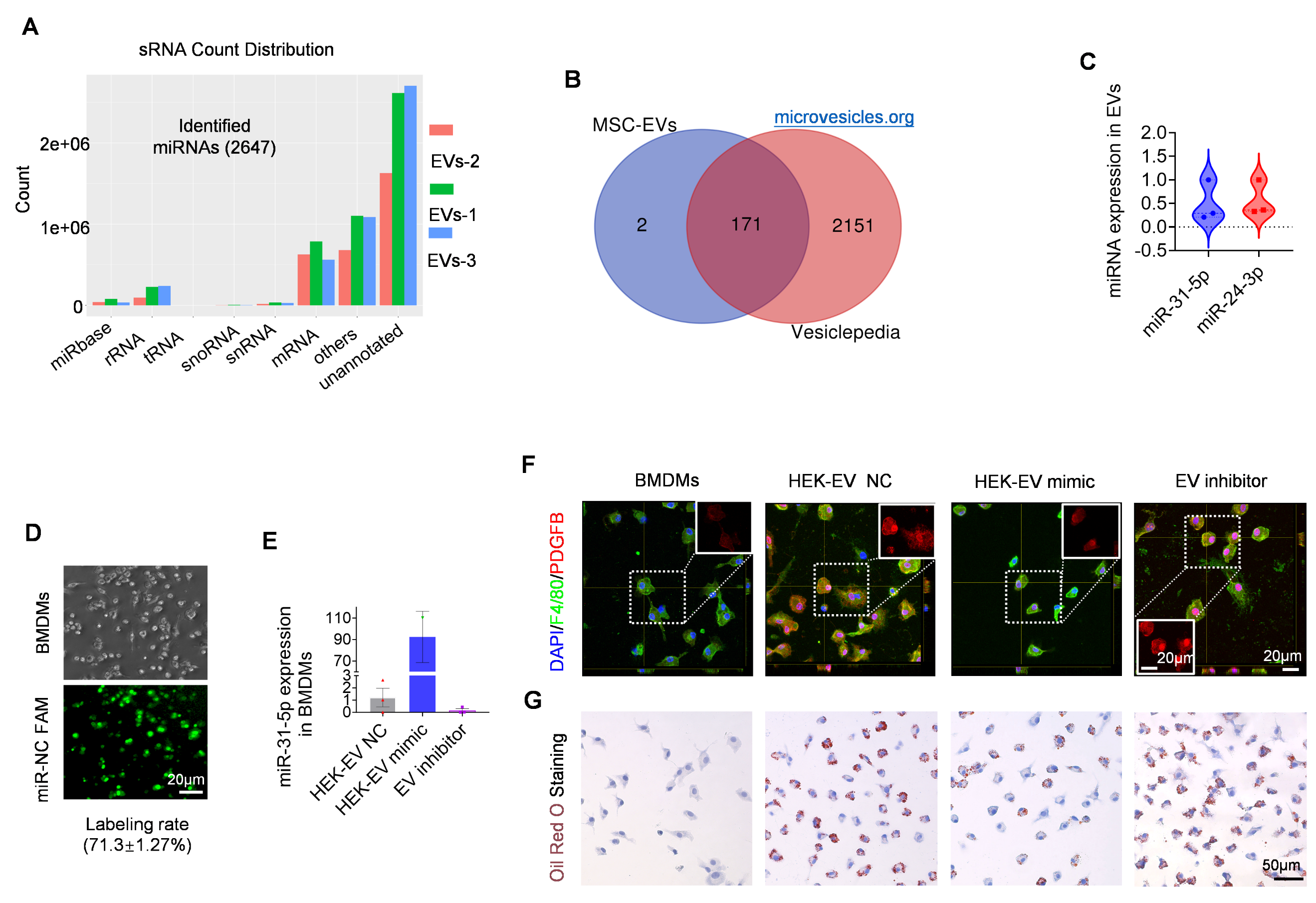


**Figure S10. Microarray profiling, EV-miRNA validation, and regulation**

(A) Microarray profiling of EVs identified 2,647 small RNAs (sRNAs), including miRNAs, across three independent EV samples (EVs-1, EVs-2, EVs-3). A notable proportion of detected RNAs were unannotated, indicating the diversity of EV-derived RNAs. (B) Venn diagram comparing MSC-derived EV miRNAs with Vesiclepedia shows an overlap of 173 conserved miRNAs across all three samples, with 171 (98.8%) also present in Vesiclepedia, underscoring the reliability of the miRNA dataset. (C) Violin plots demonstrate stable and reproducible levels of miR-31-5p and miR-24-3p across the three EV samples, validating the consistency of the miRNA profiling experiments. (D) Fluorescence microscopy of bone marrow-derived macrophages (BMDMs) transfected with 6-Carboxyfluorescein (FAM)-labeled miRNA negative control (miR-NC) confirmed a transfection efficiency of 71.3 ± 1.27% (n = 8). Scale bar: 20 μm.

(E) Bar graph showing miR-31-5p expression in BMDMs treated with HEK-derived EVs transfected with miRNA negative control (HEK-EV NC), miR-31-5p mimic (HEK-EV mimic), or MSC-derived EVs transfected with miR-31-5p inhibitor (MSC-EV inhibitor). Effective delivery and functional regulation of miR-31-5p in recipient cells were confirmed (n = 3). (F) Representative immunofluorescence images of PA-stimulated BMDMs treated with modified EVs ((HEK-EV NC, HEK-EV mimic, or EV inhibitor) show significant differences in PDGFB expression (red), with F4/80-positive staining (green) marking BMDMs. Insets highlight PDGFB changes in single cells. Scale bar: 20 μm. (G) Oil Red O staining of PA-treated BMDMs demonstrates distinct changes in lipid droplet accumulation following treatment with modified EVs, quantification showing in Fig. 6G. Scale bar: 50 μm. Data are mean ± SEM (C–E). “n” denotes the number of biological replicates (C) and experimental replicates (E).
